# miR-4653-3p overexpression is associated with a poor prognosis of pancreatic ductal adenocarcinoma via HIPK2 downregulation

**DOI:** 10.1038/s41598-022-22950-2

**Published:** 2022-10-26

**Authors:** Kenichi Hirabayashi, Masaki Miyazawa, Yumi Takanashi, Masashi Morimachi, Aya Kawanishi, Tsubasa Saika, Toshio Nakagohri, Naoya Nakamura

**Affiliations:** 1 Department of Diagnostic Pathology, Faculty of Medicine, University of Toyama, 2630 Sugitani, Toyama, 930-0194 Japan; 2 Department of Pathology, Tokai University School of Medicine, 143 Shimokasuya, Isehara, Kanagawa 259-1193 Japan; 3 Department of Health Management, Tokai University, Hiratsuka, Kanagawa 259-1292 Japan; 4 Department of Gastroenterology and Hepatology, Tokai University School of Medicine, 143 Shimokasuya, Isehara, Kanagawa 259-1193 Japan; 5 Division of Diagnostic Pathology, Tokai University Hospital, 143 Shimokasuya, Isehara, Kanagawa 259-1193 Japan; 6 Department of Surgery, Tokai University School of Medicine, 143 Shimokasuya, Isehara, Kanagawa 259-1193 Japan

**Keywords:** Cancer, Gastrointestinal cancer, miRNAs

## Abstract

Pancreatic ductal adenocarcinoma (PDAC) is a lethal malignant tumor. Several upregulated and downregulated microRNAs (miRNAs) are associated with invasiveness, tumorigenesis, and prognosis of PDAC. Herein, using in situ hybridization, we evaluated miR-4653-3p expression and pancreatic intraepithelial neoplasia (PanIN) and the association between miR-4653-3p expression and clinicopathological factors in PDAC patients. The miR-4653-3p target was also identified. Ninety PDAC cases, including 30 each with normal pancreatic ducts, low-grade PanINs, and high-grade PanINs, were evaluated. miR-4653-3p expression increased in the order—normal pancreatic duct, low-grade PanIN, high-grade PanIN, and PDAC—with no expression detected in normal pancreatic duct. High expression significantly correlated with advanced pathological T stage, lymph node metastasis, advanced Union for International Cancer Control stage, perineural invasion, venous involvement, and shorter overall and disease-specific survival. Homeodomain Interacting Protein Kinase 2 (HIPK2) was identified as a miR-4653-3p target based on mRNA microarray analysis and database screening. In MIA PaCa-2 cells, miR-4653-3p significantly downregulated HIPK2 expression. HIPK2 expression, unlike that of miR-4653-3p, decreased in the order—normal pancreatic duct, low-grade PanIN, high-grade PanIN, and PDAC. Low HIPK2 expression was associated with shorter overall and disease-specific survival in PDAC patients. Thus, miR-4653-3p associates with tumorigenesis and worse prognosis, partly by reducing HIPK2 expression.

## Introduction

Pancreatic ductal adenocarcinoma (PDAC) is a lethal malignant tumor. It has few specific symptoms, is usually diagnosed at an advanced stage, and only approximately 10% of cases are operable^1^. Pancreatic intraepithelial neoplasia (PanIN) is a microscopically flat or papillary intraductal lesion, considered the main precursor lesion of invasive ductal carcinoma^2^. PanIN was initially classified into three grades, PanIN-1 (PanIN-1A and 1B), PanIN-2, and PanIN-3^3^, but has since been reclassified as either low-grade (PanIN-1 and PanIN-2) or high-grade (PanIN-3) PanIN^2,4^.

MicroRNAs (miRNAs) are endogenous, non-coding RNA molecules, typically 18–22 nucleotides in length, that play important roles in biological processes, such as apoptosis, metabolism, cell growth, and differentiation, by negatively modulating gene expression at the post-transcriptional level^5,6^. Furthermore, the role of miRNAs as oncogenic or tumor suppressors has been reported in various cancers, including pancreatic cancer^6^. Multiple miRNAs are involved in PDAC, including two types that are involved in PDAC development^7^. Some miRNAs are upregulated in tumor cells and are classified as potent oncogenes, while others are downregulated and classified as tumor suppressors^7^. For instance, miR-21 is classified as an oncogene because its overexpression increases the proliferation, invasion, and chemoresistance of pancreatic cancer cells to gemcitabine^8^. In contrast, miR-96 is classified as a tumor suppressor because it decreases invasion and migration of cancer cells, delaying tumor growth through the downregulation of *KRAS*^9^. Both upregulation and downregulation of miRNAs have also been reported in PanIN. For instance, miR-148a, miR-217, and miR-145 are downregulated, whereas miR-10b and miR-196 are upregulated in PanIN II–III compared with that in normal pancreas^6,10,11^.

miR-4653-3p is a mature miRNA. Its upregulation has been reported in xanthohumol-treated glioblastoma cells^12^, in glioma stem cells compared with that in neural stem cells^13^, and in breast cancer MCF-7 cells overexpressing the HER-2 gene^14^. Moreover, miR-4653-3p downregulation has been reported in paclitaxel-resistant nasopharyngeal carcinoma CNE-1/Taxol cells compared with that in parental CNE-1 cells^15^ and in recurrence/metastatic lesions compared with that in primary lesions in patients with breast cancer who relapsed after tamoxifen treatment^16^. However, to the best of our knowledge, the expression of miR-4653-3p in PDAC patients has not been investigated, as of date.

In this study, we investigated the association between miR-4653-3p expression, as determined via in situ hybridization (ISH), and clinicopathological factors and assessed the impact of miR-4653-3p expression on survival outcomes in patients with PDAC. Furthermore, we identified the target of miR-4653-3p, which is associated with survival outcomes, and found that miR-4653-3p downregulates HIPK2 and that it may be associated with metastasis, shorter survival, and tumorigenesis in PDAC.

## Results

### Clinicopathological features

The mean age of the 90 patients with PDAC (43 males, 47.8%; 47 females, 52.2%) was 68 years (43–86 years) (Table 1). The mean tumor size was 37 mm (7–110 mm) in diameter. Peripheral nerve invasion, lymphatic involvement, and venous involvement were found in 81 (90%), 79 (87.8%), and 84 (93.3%) cases, respectively. G1 (well-differentiated adenocarcinoma), G2 (moderately differentiated adenocarcinoma), and G3 (poorly differentiated adenocarcinoma) tumors were diagnosed in 44 (48.9%), 44 (48.9%), and 2 (2.2%) patients, respectively. pT1 (tumor size ≤ 2 cm), pT2 (tumor size > 2 cm but ≤ 4 cm), and pT3 (tumor size > 4 cm) cancers were diagnosed in 18 (20%), 43 (47.8%), and 29 (32.2%) patients, respectively. No patient was diagnosed with pT4 (tumor involving the celiac axis, superior mesenteric artery, or common hepatic artery) cancer. pN1 (metastasis in 1–3 nodes) and pN2 (metastasis in ≥ 4 nodes) cancers were diagnosed in 32 (35.6%) and 17 (18.9%) patients, respectively. Four patients (4.4%) had distant metastases. Stages IA, IB, IIA, IIB, III, and IV cancers were diagnosed in 13 (14.4%), 19 (21.1%), 9 (10%), 31 (34.4%), 14 (15.6%), and 4 (4.4%) patients, respectively.

**Table 1:** miR-4653-3p expression and clinicopathological parameters.

Total number of cases	miR4653-3p expression		*p *value
	Low	High	
(*n* = 90)	(*n* = 32)	(*n* = 58)	
	*n* (%)	*n* (%)	
**Age**			
≤ 65 years (*n* = 32)	13 (40.6)	19 (59.4)	0.456
> 65 years (*n* = 58)	19 (32.8)	39 (67.2)	
**Gender**			
Male (*n* = 43)	12 (27.9)	31 (72.1)	0.147
Female (*n* = 47)	20 (42.6)	27 (57.4)	
**Primary tumor (pT)**			
pT1-pT2 (*n* = 61)	28 (45.9)	33 (54.1)	0.003
pT3 (*n* = 29)	4 (13.8)	25 (86.2)	
**Regional lymph node metastasis (pN)**			
pN0 (*n* = 41)	20 (48.8)	21 (51.2)	0.017
pN1-2 (*n* = 49)	12 (24.5)	37 (75.5)	
**Distant metastasis (pM)**			
pM0 (*n* = 86)	31 (36)	55 (64)	0.552
pM1 (*n* = 4)	1 (25)	3 (75)	
**Stage**			
Stage IA–IB (*n* = 32)	19 (59.4)	13 (40.6)	< 0.001
Stage IIA–IV (*n* = 58)	13 (22.4)	45 (77.6)	
**Perineural invasion**			
No (*n* = 9)	6 (66.7)	3 (33.3)	0.048
Yes (*n* = 81)	26 (32.1)	55 (67.9)	
**Lymphatic involvement**			
No (*n* = 11)	6 (54.5)	5 (45.59	0.143
Yes (*n* = 79)	26 (32.9)	53 (67.1)	
**Venous involvement**			
No (*n* = 6)	5 (83.3)	1 (16.7)	0.02
Yes (*n* = 84)	27 (32.1)	57 (67.9)	
**Tumor grade**			
G1 (*n* = 44)	15 (34.1)	29 (65.9)	0.776
G2 or G3 (*n* = 46)	17 (37)	29 (63)	

### Analysis of miR-4653-3p expression using ISH and its correlation with clinicopathological factors

**Figure 1:**
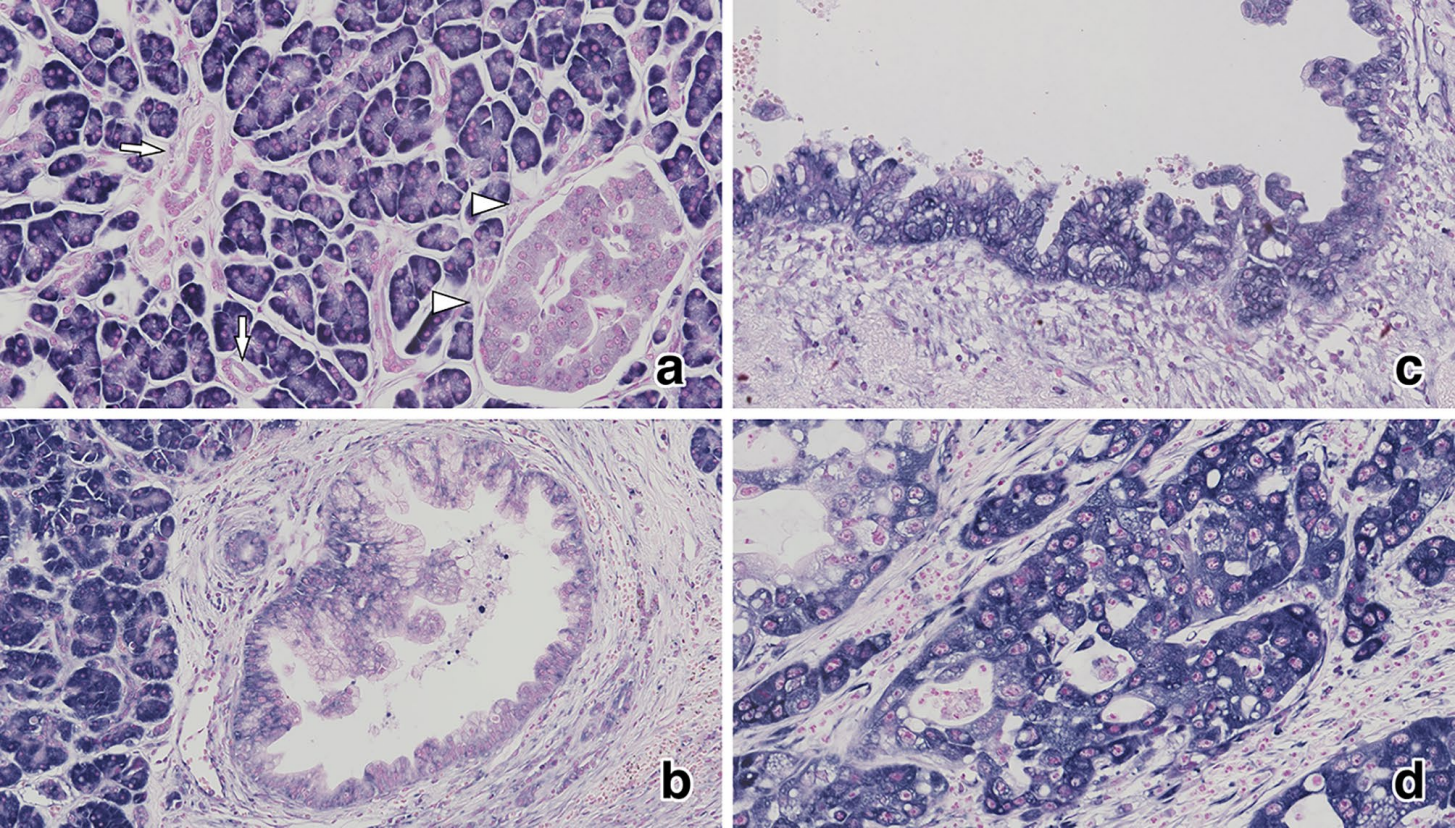
In situ miR-4653-3p hybridization. Normal pancreatic tissue showed that acinar cells were diffusely and strongly positive for miR-4653-3p expression, while pancreatic ducts (arrow) and islet cells (arrowhead) were negative and weakly positive for miR-4653-3p expression, respectively (**a**). Low-grade pancreatic intraepithelial neoplasms (PanINs) were weakly and focally positive for miR-4653-3p expression (**b**). High-grade PanINs (**c**) and pancreatic ductal adenocarcinomas (**d**) were diffusely and strongly positive for miR-4653-3p expression.

**Figure 2:**
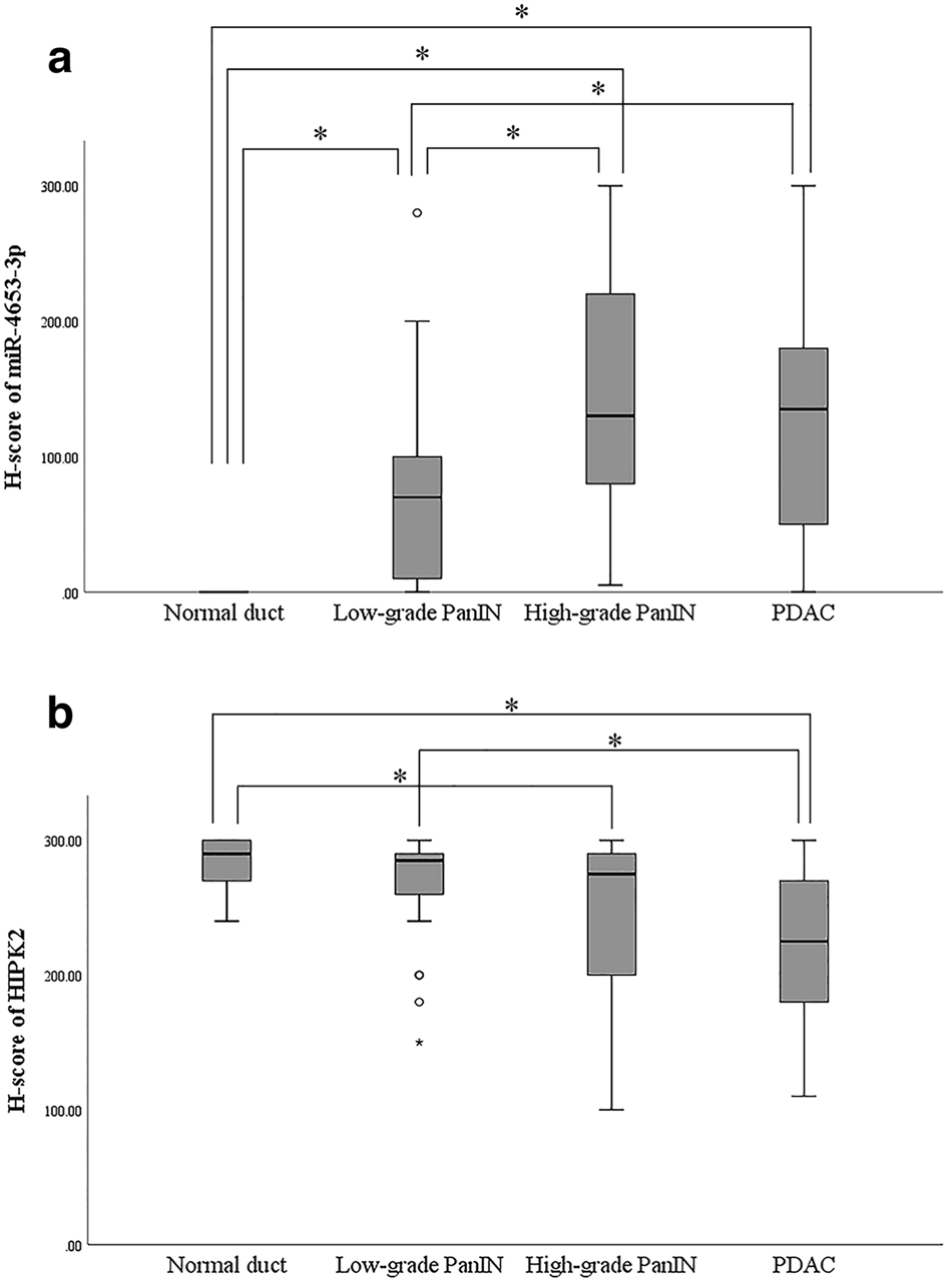
H-score of in situ miR-4653-3p hybridization and HIPK2 immunohistochemistry. miR-4653-3p was not expressed in normal pancreatic ducts, and its expression increased in the order normal pancreatic duct < low-grade PanIN < high-grade PanIN < PDAC (**a**). In contrast, HIPK2 expression decreased gradually, in the order normal pancreatic duct, low-grade PanIN, high-grade PanIN, and PDAC (**b**). *represents significant differences in miR-4653-3p expression and HIPK2 H-scores (*p* < 0.05).

**Figure 3:**
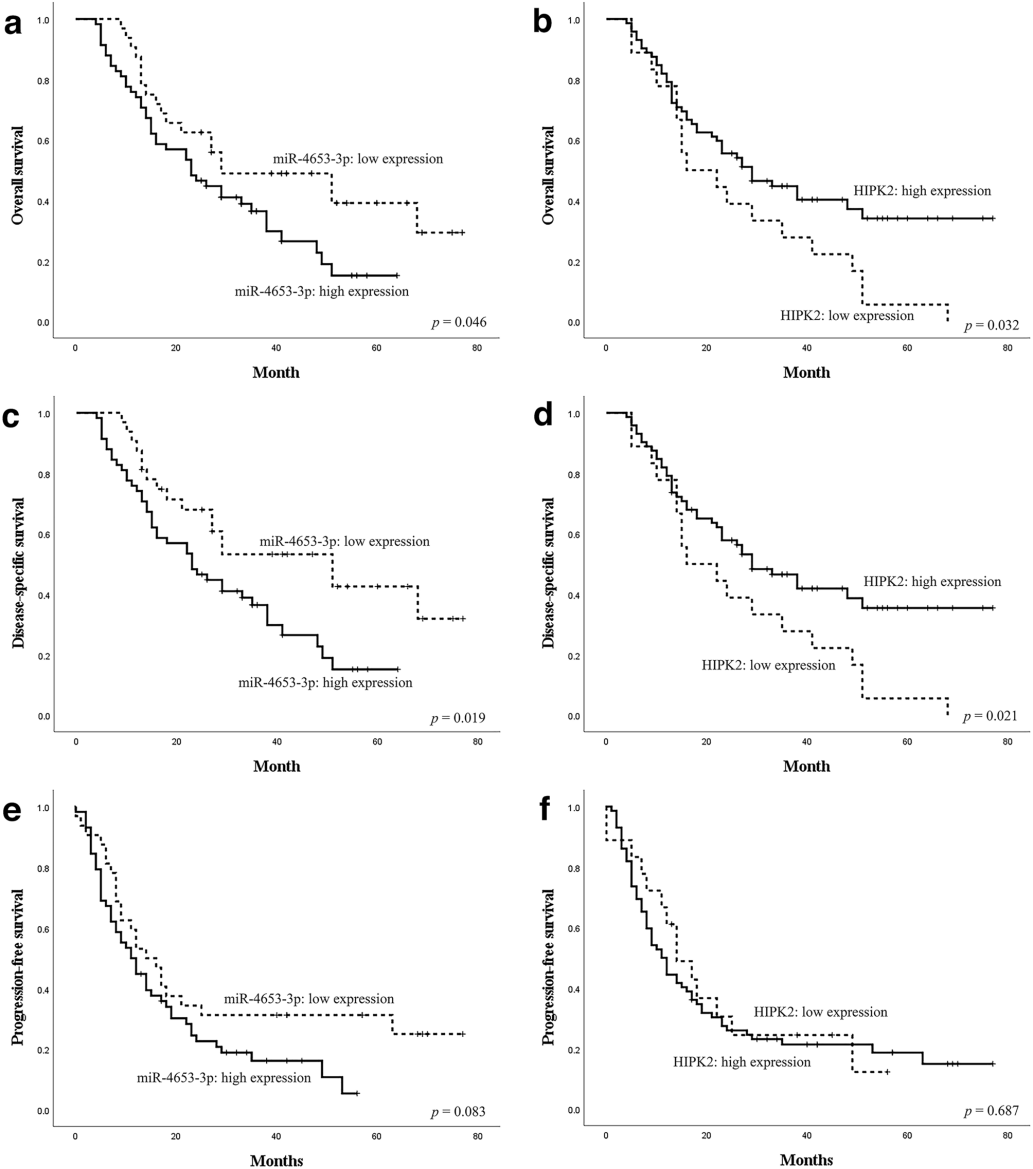
Survival analysis of patients with pancreatic ductal adenocarcinoma in relation to the expression of miR-4653-3p and HIPK2. High miR-4653-3p expression was associated with shorter overall (**a**) and disease-specific survival (**c**). In contrast, low HIPK2 expression was associated with shorter overall (**b**) and disease-specific survival (**d**). There was no significant difference in the association between progression-free survival and the expression of miR-4653-3p (**e**) and HIPK2 (**f**).

miR-4653-3p was diffusely and strongly expressed in the cytoplasm of acinar cells in normal pancreatic tissues. Islet cells expressed either little or no miR-4653-3p (Fig. 1a). However, normal pancreatic ducts did not express miR-4653-3p (H-score 0, *n* = 30) (Figs. 1a and 2a). The mean H-score was 67.7 (0–280) in low-grade PanIN, 143.3 (5–300) in high-grade PanIN, and 127.9 (0–300) in PDAC (Figs. 1b–d and 2a). There were significant differences in the mean miR-4653-3p H-scores between PDAC and normal pancreatic duct (*p* < 0.001), PDAC and low-grade PanIN (*p* < 0.001), high-grade PanIN and normal pancreatic duct (*p* < 0.001), high-grade PanIN and low-grade PanIN (*p* = 0.001), and low-grade PanIN and normal pancreatic duct (*p* < 0.001). However, there was no significant difference in the mean miR-4653-3p H-scores between high-grade PanIN and PDAC (*p* = 0.397). In PDAC, 32 cases (35.6%) had low miR-4653-3p expression while 58 cases (64.4%) had high miR-4653-3p expression. High miR-4653-3p expression was associated with pT3 (tumor size > 4 cm; *p* = 0.003), pN1-2 (*p* = 0.017), perineural invasion (*p* = 0.048), venous involvement (*p* = 0.02), and stages IIA–IV (*p* < 0.001). There were no correlations between miR-4653-3p expression and age, sex, pM-factor, lymphatic involvement, or tumor grade (Table 1). High miR-4653-3p expression was associated with shorter overall and disease-specific survival in the log-rank test. The median overall survival was 29 months (95% CI: 3.75–54.25) for patients with low miR-4653-3p expression and 23 months (95% CI: 14.522–31.478) for patients with high miR-4653-3p expression (log-rank test: *p* = 0.046, Fig. 3a). The median disease-specific survival was 51 months (95% CI: 25.176–76.824) for patients with low miR-4653-3p expression and 23 months (95% CI: 14.522–31.478) for patients with high miR-4653-3p expression (log-rank test: *p* = 0.019, Fig. 3c). However, multivariate analysis showed that miR-4653-3p was not an independent prognostic factor (Supplementary Table 1a). There was no significant difference in progression-free survival between low and high miR-4653-3p expression groups (log-rank test: *p* = 0.083, Fig. 3e).

### mRNA microarray

To identify the target of miR-4653-3p, we performed mRNA microarray analysis and compared mRNA expression between MIA PaCa-2 cells transfected with miR-4653-3p mimic and negative control cells. mRNA microarray analysis revealed that 517 mRNAs were significantly downregulated, and 635 mRNAs were significantly upregulated in MIA PaCa-2 cells transfected with miR-4653-3p mimic compared with that in negative control cells (|fold change|> 2.0, *p* < 0.05) (Supplementary Table 2).

### Screening miR-4653-3p target molecules

TargetScan v.7.2^17^ (http://www.targetscan.org/vert_72/) and Human Protein Atlas v. 19.3^18^ (https://www.proteinatlas.org/) were used to screen for targets of miR-4653-3p. First, we identified 163 out of the 517 mRNAs downregulated in miR-4653-3p-transfected cells by microarray analysis and listed them as predicted targets of miR-4653-3p in TargetScan. Next, among these 163 mRNAs, we selected 13 mRNAs (ADAL, ADCK1, BCORL1, GNPTG, HIPK2, LRRN3, NCOA1, NKAIN1, PLAGL2, POMGNT1, PXK, TUBD1, and ZKSCAN1) showing significantly unfavorable prognosis with low mRNA expression in pancreatic cancer, which were expressed in normal pancreatic ducts in the Human Protein Atlas. In the above screening, we focused on HIPK2 because low HIPK2 expression is associated with poor prognosis in pancreatic cancer, and HIPK2 is associated with tumor suppressor p53, one of the major molecules underlying PDAC tumorigenesis.

### Luciferase reporter assay, reverse transcription-quantitative polymerase chain reaction (RT-qPCR), and western blot analysis

**Figure 4:**
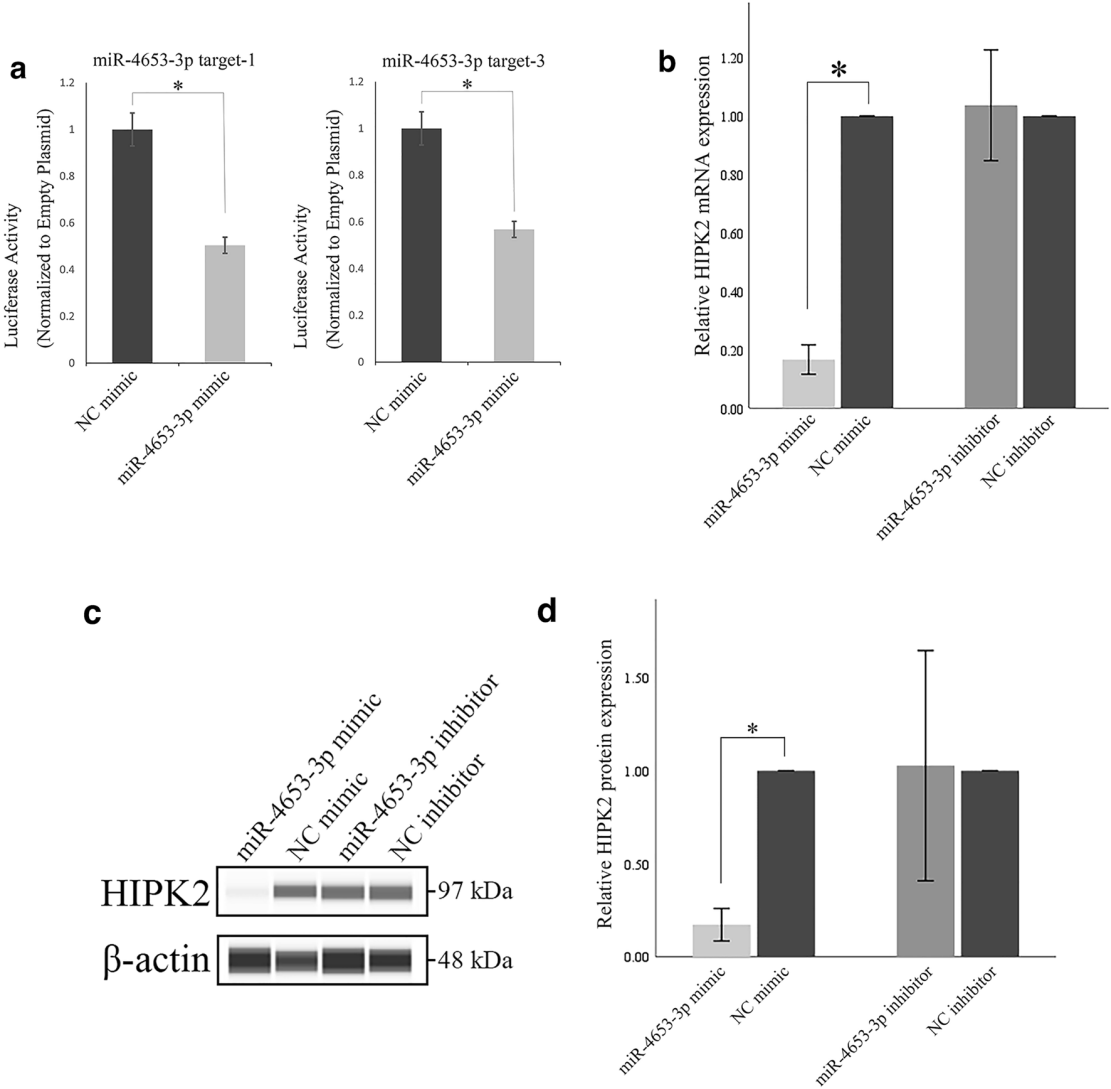
Luciferase reporter assay, RT-qPCR, and western blot analysis. Luciferase reporter assay showed that the miR-4653-3p mimic downregulated luciferase activity on miR-4653-3p target positions compared with the negative control (NC) mimic (**a**). In real-time RT-qPCR (**b**), the expression of HIPK2 mRNA in PDAC cells transfected with miR-4653-3p mimic was downregulated compared with the expression in NC cells. In contrast, there was no significant difference in HIPK2 mRNA expression between PDAC cells transfected with miR-4653-3p inhibitor and NC cells. In the western blot analysis (**c**, **d**), the expression of HIPK2 in PDAC cells transfected with the miR-4653-3p mimic was downregulated compared with that in NC cells. In contrast, there was no significant difference in HIPK2 mRNA expression between PDAC cells transfected with miR-4653-3p inhibitor and NC cells. Figure 4c shows the HIPK2 and β-actin areas cut out from the original gel image for clarity. Original chemiluminescent image of the capillary and charge data in the western blot analysis are presented in Supplementary Fig. 2. *p < 0.05.

To determine whether miR-4653-3p could downregulate HIPK2 expression, we performed real-time RT-qPCR, western blot, and luciferase assays. The miR-4653-3p mimic downregulated luciferase activity to 50.0% and 43.2% at miR-4653-3p target position 929–935 (target 1) and 7620–7627 (target 3) compared with that in the negative control (NC) mimic, but not at target position 5947–5953 (target 2; *p* = 0.500). Means ± (standard deviation) SD from four independent experiments are shown in Fig. 4a. In real-time RT-qPCR, HIPK2 mRNA expression in PDAC cells transfected with miR-4653-3p mimic was downregulated to 16.6% compared with that in NC cells (*p* < 0.001). In contrast, there was no significant difference in the expression of HIPK2 mRNA between PDAC cells transfected with miR-4653-3p inhibitor and NC cells (*p* = 0.683, Fig. 4b). In the western blot assay, the expression of HIPK2 in PDAC cells transfected with miR-4653-3p mimic was downregulated to 17.3% compared with that in NC cells (*p* < 0.001). In contrast, there was no significant difference in the expression of HIPK2 mRNA between PDAC cells transfected with miR-4653-3p inhibitor and NC cells (*p* = 0.935; Fig. 4c, d). Taken together, these results show that HIPK2 is the target of miR-4653-3p and that miR-4653-3p downregulates HIPK2 in PDAC cell lines.

### HIPK2 immunohistochemistry

**Figure 5:**
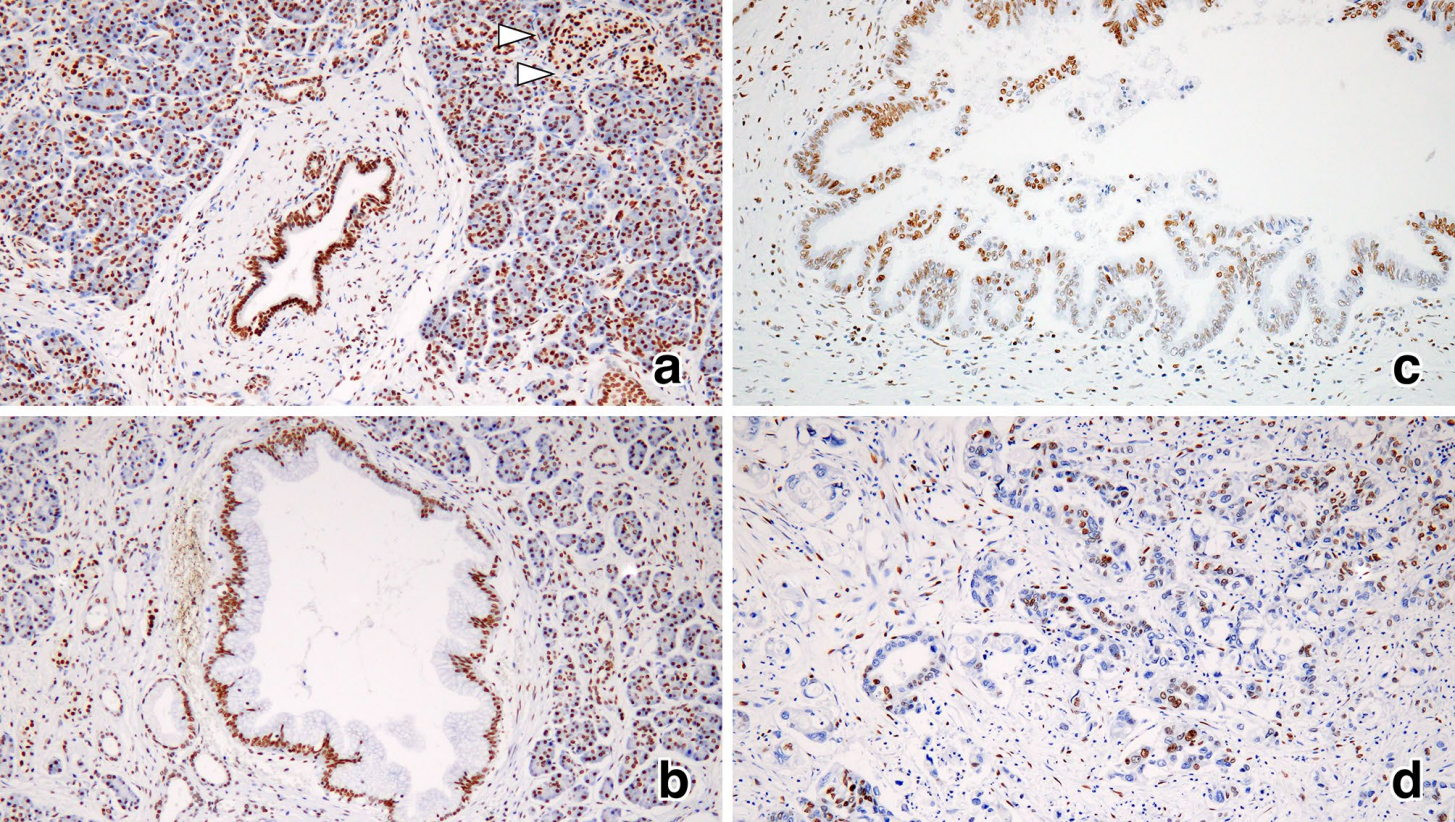
Immunohistochemical analysis of HIPK2 expression. HIPK2 was expressed in nuclei in pancreatic ducts, acinar cells, and islet cells in normal pancreatic tissue (**a**) and in low-grade PanIN (**b**). In high-grade PanIN (**c**) and PDAC (**d**), HIPK2 was focally negative or weakly expressed.

**Figure 6:**
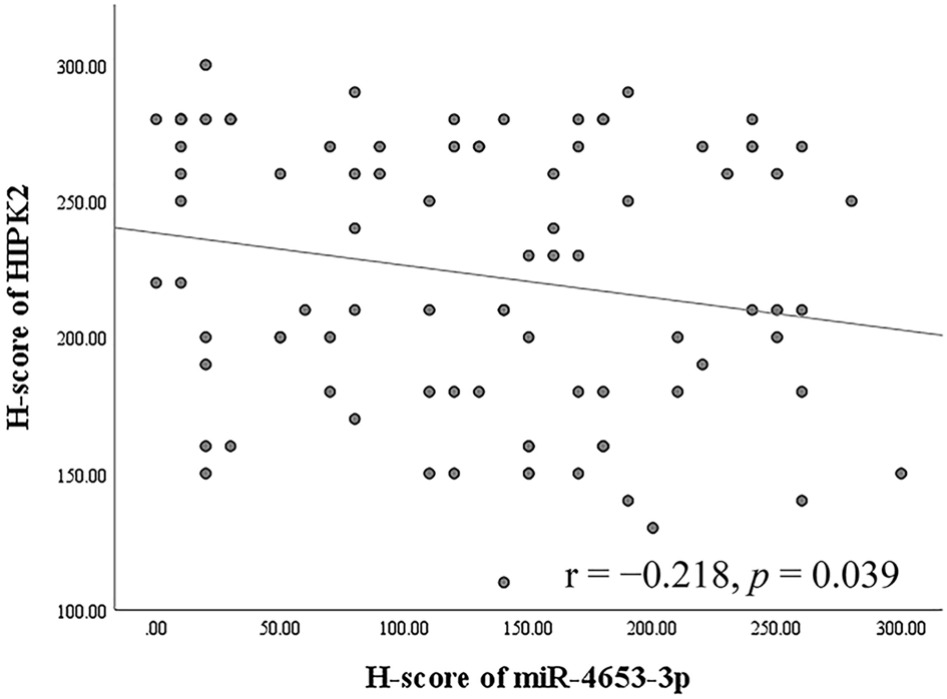
Correlation between HIPK2 and miR-4653- 3p expression. There was a weak inverse correlation between the H-score of immunohistochemistry of HIPK2 and the H-score of in situ hybridization of miR-4653-3p in PDAC (r =  − 0.218, *p* = 0.039).

Next, immunohistochemistry was performed to detect HIPK2. HIPK2 was expressed in the nuclei of normal pancreatic ducts, normal acinar cells, normal islet cells, PanIN cells, and PDAC cells (Fig. 5). The mean H-score for HIPK2 was 282.3 (240–300) in patients with normal pancreatic ducts, 267 (150–300) in patients with low-grade PanIN, 240.5 (100–300) in patients with high-grade PanIN, and 223.1 (110–300) in patients with PDAC (Fig. 2b). Significant differences were observed in the mean HIPK2 H-scores between normal pancreatic duct and PDAC (*p* < 0.001), low-grade PanIN and PDAC (*p* < 0.001), and normal pancreatic duct and high-grade PanIN (*p* = 0.003). However, no significant differences in the mean H-scores of HIPK2 between low-grade PanIN and high-grade PanIN (*p* = 0.077), high-grade PanIN and PDAC (*p* = 0.213), and normal pancreatic duct and low-grade PanIN (*p* = 0.064) were observed. Among the patients with PDAC, 18 (20%) had low HIPK2 expression, while 72 (80%) had high HIPK2 expression. There was a weak inverse correlation between the H-score of immunohistochemistry of HIPK2 and the H-score of ISH of miR-4653-3p in PDAC (r =  − 0.218, *p* = 0.039) (Fig. 6). Low HIPK2 expression was associated with shorter overall and disease-specific survival in the log-rank test. The median overall survival was 16 months (95% CI: 1.448–30.552) for patients with low HIPK2 expression and 29 months (95% CI: 18.209–39.791) for patients with high HIPK2 expression (log-rank test: *p* = 0.032, Fig. 3b). The median disease-specific survival was 16 months (95% CI: 1.448–30.552) for patients with low HIPK2 expression and 29 months (95% CI: 19.118–38.882) for patients with high HIPK2 expression (log-rank test: *p* = 0.021, Fig. 3d). However, multivariate analysis showed that miR-HIPK2 was not an independent prognostic factor (Supplementary Table 1b). There was no significant difference in progression-free survival between low and high HIPK2 expression groups (log-rank test: *p* = 0.687, Fig. 3f). There were no associations with other clinicopathological factors, except for overall and disease-specific survival (Supplementary Table 3).

## Discussion

In this study, we used ISH to evaluate the miR-4653-3p expression in patients with PDAC and its correlation with clinicopathological factors. High miR-4653-3p expression significantly correlated with advanced pT, lymph node metastasis, advanced UICC stage, perineural invasion, venous involvement, and shorter overall and disease-specific survival. In addition, miR-4653-3p was not expressed in normal pancreatic ducts, and its expression increased in the order—normal pancreatic duct < low-grade PanIN < high-grade PanIN < PDAC. These results suggest that miR-4653-3p is involved in the early stages of PDAC tumorigenesis in the normal pancreatic duct, low-grade PanIN, and high-grade PanIN. Furthermore, miR-4653-3p reduced HIPK2 expression in vitro, and miR-4653-3p expression inversely correlated with HIPK2 expression in vivo. Unlike that of miR-4653-3p, HIPK2 expression decreased gradually in the order—normal pancreatic duct, low-grade PanIN, high-grade PanIN, and PDAC. In addition, low HIPK2 expression was associated with shorter and disease-specific survival in patients with PDAC. These results indicate that miR-4653-3p is associated with tumorigenesis and a shorter prognosis, partly by reducing HIPK2 expression.

HIPK2 is a nuclear serine/threonine kinase that represses the transcription of homeodomain-containing transcription factors^19^. Its expression and role have been reported in a variety of tumors. HIPK2 is downregulated in tumor cells compared with normal tissues in hepatocellular carcinoma^20^, esophageal squamous cell carcinoma^21^, urinary bladder cancer^22^, colorectal cancer^23^, and pancreatic cancer^24^. In these tumors, the downregulation of HIPK2 was associated with poor prognosis, promoted migration, tumor growth, metastasis, or increased cell viability^20–26^. In contrast, HIPK2 expression was upregulated in tumor cells in tonsillar squamous cell carcinoma^27^ and cervical cancer^28,29^. In tonsillar squamous cell carcinoma, HIPK2 overexpression was associated with shorter overall survival and disease-free survival^27^. The expression and role of HIPK2 may differ depending on tumor site and histological type.

The expression of HIPK2 in pancreatic tissues, PanIN, and PDAC has been reported in several studies. Qin et al. reported higher HIPK2 expression in adjacent normal pancreatic tissue compared with that in pancreatic cancer tissue, and low HIPK2 expression was associated with poor prognosis^23^. Valente et al. reported that HIPK2 expression was significantly lower in PanIN-3 (currently classified as high-grade PanIN) and PDAC than in normal tissues^30^. These reports and our results of HIPK2 expression support the notion that HIPK2 downregulation may be associated with the tumorigenesis of PDAC.

We hypothesize that the association between HIPK2 and p53 may also play an important role in the prognosis of pancreatic cancer. The tumor suppressor p53 interacts with HIPK2. HIPK2-mediated phosphorylation of p53 at Ser46 enhances p53-induced apoptosis^31–33^. HIPK2 may stabilize p53 by downregulating p53-induced Mdm2 protein. p53 is one of the major genes associated with tumorigenesis and prognosis of PDAC^34^. Therefore, downregulation of HIPK2 in pancreatic cancer leads to the downregulation of p53-associated tumor suppressors, resulting in tumorigenesis and poor prognosis. The inactivation of p53 occurs late in the development of pancreatic neoplasia, for example, in high-grade PanIN and PDAC, but not in low-grade PanIN^35^. Based on our results and the HIPK2 expression pattern reported previously, we hypothesized that HIPK2 might be associated with the late stage of PDAC tumorigenesis, which involves p53. In contrast, miR-4653-3p expression was significantly higher in the normal pancreatic duct than in low-grade PanIN. These results indicate that other molecules, besides HIPK2, are controlled by miR-4653-3p and are associated with PDAC tumorigenesis.

In conclusion, this study shows that miR-4653-3p might promote the tumorigenesis and invasiveness of PDAC, in part by inhibiting the expression of HIPK2. ISH of miR-4653-3p may prove useful in predicting tumor invasiveness and prognosis of PDAC. However, more complex molecular mechanisms and other target molecules may also be associated with PDAC tumorigenesis. Thus, further studies are warranted to clarify the association between target molecules and miR-4653-3p in PDAC tumorigenesis.

## Materials and methods

### Case selection

Ninety PDAC cases were examined. All patients were diagnosed and treated at Tokai University Hospital (Kanagawa, Japan) between March 2000 and October 2013. Patients who underwent preoperative chemotherapy were excluded from the study. Formalin-fixed and paraffin-embedded (FFPE) tissue samples were cut into 4 μm-thick sections and stained with hematoxylin and eosin (H&E). The H&E-stained sections, pathology reports, and patient medical records were reviewed to confirm PDAC diagnosis and to establish the clinicopathological factors of the patients, including age, sex, tumor size, histological grade, lymph node metastasis, distant metastasis, tumor stage, perineural invasion, lymphatic involvement, venous involvement, and overall survival. Pathological tumor, node, and metastasis (pTNM) staging and histological grade were classified as outlined in the Union for International Cancer Control, 8th edition^36^. Overall survival was defined as the time interval between surgery and death or the date of the last patient visit. In addition, 30 normal pancreatic ducts, 30 low-grade PanINs, and 30 high-grade PanINs among the PDAC specimens were also examined.

### ISH of miR-4653-3p

ISH was performed on 4 μm-thick sections of FFPE tissues obtained from PDAC patients. Sections were deparaffinized in xylene and rehydrated in decreasing concentrations of ethanol diluted in PBS. The slides were incubated in proteinase K solution (15 μg/mL) at 37 °C for 10 min and then washed with PBS. A hybridization mix containing digoxigenin-labeled LNA™ microRNA probe (5DigN/TCTCCAAGCAACCCTTAACTCCA/3DigN, final concentration: 5 nM, EXIQON, Woburn, MA, USA) was applied to the sections and hybridization was allowed to occur at 54 °C for 60 min. Thereafter, slides were washed with SSC buffer and incubated with a blocking solution (2% normal sheep serum/PBS, 0.1% Tween20, 1% BSA) for 15 min in a humidified chamber. The blocking solution was removed, anti-DIG reagent, polyclonal sheep anti-DIG-AP diluted 1:800 with antibody diluent (Roche Diagnostics, Mannheim, Germany) were applied, and the sections incubated for 60 min at room temperature. After washing with PBS-T, the cells were incubated with AP substrate (NBT-BCIP (DAKO, Carpinteria, CA, USA) + 0.2 mM levamisole) for 2 h at 30 °C in a humidified chamber. Slides were incubated twice for 5 min in KTBT buffer to stop the reaction and then washed with water before applying Nuclear Fast Red™ (ScyTek, Logan, UT, USA) for 2 min for nuclear counterstaining. Finally, the slides were washed with water and dehydrated in an ethanol solution.

### Cell culture

The pancreatic adenocarcinoma cell line, MIA PaCa-2, was obtained from the American Type Culture Collection (ATCC; Manassas, VA, USA). Cells were cultured in Dulbecco’s modified Eagle’s medium (DMEM, Gibco) containing 10% fetal bovine serum and then incubated at 37 °C in a humidified atmosphere containing 5% CO_2_.

### Transfection of miRNA mimic and inhibitor

miRIDIAN miRNA mimic and hairpin inhibitor (Dharmacon, Lafayette, CO, USA) of miR-4653-3p were transfected into MIA PaCa-2 cells using DharmaFECT 2 Transfection Reagent (Dharmacon) according to the manufacturer’s protocols. miRIDIAN miRNA Mimic Negative Control #1 (Dharmacon) and miRIDIAN miRNA Hairpin Inhibitor Negative Control #1 (Dharmacon) were used as miRNA mimic and inhibitor controls, respectively. The final concentration of each miRNA mimic, inhibitor, and negative control was 25 nM.

### mRNA microarray

Total RNA was extracted from MIA PaCa-2 cells transfected with miR-4653-3p mimic or its negative control using the AllPrep DNA/RNA Mini kit (Qiagen, Hilden, Germany) according to the manufacturer’s instructions. RNA samples were sent to Macrogen (Macrogen Japan, Kyoto, Japan) for mRNA microarray analysis using SurePrint G3 Human Gene Expression 8 × 60 K v3 (Agilent, Inc., Santa Clara, CA, USA). Comparative analysis between MIA PaCa-2 cells transfected with miR-4653-3p mimic (*n* = 4) and MIA PaCa-2 cells transfected with mimic negative control (*n* = 4) was carried out using an independent *t*-test. A |fold change|> 2.0 and *p* < 0.05 were set as the significance threshold. Data analysis was conducted using R 3.3.3 (www.r-project.org).

### Screening for a miR-4653-3p target molecule

TargetScan v.7.2^17^ (http://www.targetscan.org/vert_72/) and the Human Protein Atlas v. 19.3^18^ (https://www.proteinatlas.org/) databases were screened to identify the miR-4653-3p target molecule.

### RT-qPCR

Total RNA was extracted from MIA PaCa-2 cells, 48 h after transfection with miR-4653-3p mimic, miR-4653-3p inhibitor, or each NC using the AllPrep DNA/RNA Mini kit (Qiagen) according to the manufacturer’s protocols. To obtain cDNA from total RNA, reverse transcription was performed using the High Capacity cDNA Reverse Transcription Kit (Thermo Fisher Scientific, Waltham, MA, USA) and a thermal cycler (Veriti Thermal Cycler, Thermo Fisher Scientific) under the following conditions: 25 °C for 10 min, 37 °C for 120 min, 85 °C for 5 min, and a cooling step at 4 °C. To analyze HIPK2 expression, quantitative real-time PCR was performed using the TaqMan probe (Hs00179759_m1, Thermo Fisher Scientific), TaqMan Universal PCR Master Mix (Thermo Fisher Scientific), and the Step One Plus system (Thermo Fisher Scientific) under the following conditions: polymerase activation at 95 °C for 10 min, 40 cycles of denaturation at 95 °C for 15 s and annealing/extension at 60 °C for 1 min. All samples (*n* = 5 in each group) were run in triplicate and normalized to GAPDH (Hs02786624_g1, Thermo Fisher Scientific).

### Western blot analysis

Western blot analysis was performed using a simple western blot assay (ProteinSimple, Santa Clara, CA, USA). Cultured cells were lysed in cell lysis buffer containing protease inhibitors before sonication. The cell lysates were then centrifuged at 10,000 rpm for 3 min to remove the soluble material. The protein concentration of cell lysates was measured using a DC protein assay (Bio-Rad, Hercules, CA, USA). For the simple western blot assay, cell lysates were diluted to 0.4 μg/μL using 0.1 × sample buffer and 5 × Fluorescent Master Mix. The primary antibodies used were as follows: HIPK2 (ab28507, Abcam, Cambridge, United Kingdom) at 1:100 dilution and β-actin monoclonal antibody (A5441, clone AC-15, Sigma-Aldrich, St. Louis, MO, USA) at 1:4,000 as the internal control. Secondary antibodies were prepared by the manufacturer. The assay was conducted according to the manufacturer’s instructions. The assay plate and capillary cartridge were placed into a simple western machine (WES; Protein Simple). All subsequent separation, immunodetection, and analysis steps were performed automatically on the WES. The Compass software (ProteinSimple) was used to visualize the chemiluminescent image of the capillary, analyze the size or charge data, and process the results. The relative HIPK2 expression peak area was normalized to that of β-actin (*n* = 3 in each experimental group).

### Luciferase reporter assay

Approximately 500–700 bp fragments containing the miR-4653-3p target site were PCR-amplified from the 3′-UTR of human HIPK2 mRNA using the following primers: forward primer for target position 929–935—5′-catatggcagcaagtgatac-3′, reverse primer for target position 929–935—5′-attggttacatgcctatgttatatc-3′; forward primer for target position 5947–5953—5′-atgcaattctatttgtctttctc-3′, reverse primer for target position 5947–5953—5′-gctattatttacttgagagatattgc-3′; forward primer for target position 7620–7627—5′-ttgcttatagttaattctagaaagg-3′, reverse primer for target position 7620–7627—5′-tcacagtttgtgtaagataatgtatc-3′. A schematic of human HIPK2 mRNA sequences with three candidate target sites for miR-4653-3p on the 3′-UTR is shown in Supplementary Fig. 1. The PCR products were subcloned into the PmeI site on the pMIR-REPORT luciferase plasmid (Invitrogen); insert orientation and nucleotide sequences were verified by sequencing. For the luciferase activity assay, MIA PaCa-2 cells were seeded in 24-well plates one day before transfection. The miR-4653-3p target site-containing pMIR-REPORT plasmid was co-transfected with miR mimics using the transfection reagent TransIT-X2 (Mirus, Madison, WI, USA). After 24 h, cells were lysed with 1 × cell culture lysis reagent, as supplied in the Luciferase Assay System (Promega, Madison, WI, USA). Luciferase activity was measured using a GloMax 20/20 luminometer (Promega). Relative translational repression was normalized to the value of the empty pMIR-REPORT luciferase plasmid.

### Immunohistochemistry

After the FFPE specimens were cut into 4 μm-thick sections and immunohistochemistry was performed to identify HIPK2 (ab28507, rabbit polyclonal; dilution, 1:200; Abcam) using BOND-MAX (Leica Microsystems, Tokyo, Japan), according to the manufacturer’s instructions. Antigen retrieval was performed by treatment with ER1 for 20 min.

### Interpretation of immunohistochemistry and ISH data

Expression measured using immunohistochemistry and ISH was evaluated using the histochemical scoring system (H-score)^37^. H-score (0–300) was calculated as the sum of the staining intensity (0 = negative, 1 +  = weak, 2 +  = moderate, and 3 +  = strong) multiplied by the percentage of cells (0–100) expressed at each intensity. The H-score was calculated using the formula: H-score = 0 × (percentage of negative cells) + 1 × (percentage of 1 + cells) + 2 × (percentage of 2 + cells) + 3 × (percentage of 3 + cells). The optimal H-score cut-off point was determined as the score with the lowest *p*-value in the overall Kaplan–Meier survival analysis method and the log-rank test. For the miR4653-3p ISH assay, an H-score < 90 was considered low, while an H-score ≥ 90 was considered high. For the HIPK2 immunohistochemistry, an H-score < 180 was considered low, while an H-score ≥ 180 was considered high.

### Statistical analyses

Statistical analyses were conducted using SPSS v26 (IBM Corp., Armonk, NY, USA). Fisher’s exact test and Pearson’s *χ*^*2*^ test were used to analyze the relationship between clinicopathological factors and the expression of miR-4653-3p or HIPK2. Two-sample Student’s *t*-test or Welch’s *t*-test was used to compare the expression of HIPK2 or miR-4653-3p as determined with RT-qPCR, western blot, immunohistochemistry, and ISH analyses. The correlation between the expression of miR-4653-3p and HIPK2 was evaluated using Spearman correlation analysis. Survival curves were plotted using the Kaplan–Meier method and compared using a log-rank test. Survival was also evaluated in univariate and multivariate analyses using Cox proportional hazard regression models to identify independent prognostic factors. First, a univariate analysis of overall survival was performed for each clinicopathological factor. Statistically significant factors in the univariate analysis were then included in the multivariable analysis using the forward selection method (likelihood ratio); *p* < 0.05 was considered statistically significant.

### Ethics approval and consent to participate

This study was approved by the Research Ethics Committee of Tokai University School of Medicine (No. 17R275) and was performed according to the Declaration of Helsinki. The informed consent was obtained from all subjects through an opt-out methodology.

## Supplementary Information

Supplementary Information.

## Data Availability

All data analyzed in this study are included in this published article and its supplementary information files. The datasets of gene expression generated and/or analyzed during the current study are available in the ArrayExpress repository, ArrayExpress accession E-MTAB-11813.
