# Supplementary material for: miR-4653-3p overexpression is associated with a poor prognosis of pancreatic ductal adenocarcinoma via HIPK2 downregulation
Source: Sci Rep. 2022 Oct 26;12:17927. doi: 10.1038/s41598-022-22950-2 (PMC9606280; doi:10.1038/s41598-022-22950-2)
Supplement: Supplementary file 1 — Supplementary Information. [file 41598_2022_22950_MOESM1_ESM.pdf]

Supplementary Information

miR-4653-3p overexpression is associated with a poor prognosis of pancreatic ductal adenocarcinoma via HIPK2 downregulation

Kenichi Hirabayashi, Masaki Miyazawa, Yumi Takanashi, Masashi Morimachi, Aya Kawanishi, Tsubasa Saika, Toshio Nakagohri, Naoya Nakamura

Supplementary Figure 1. Schematic showing HIPK2 mRNA with three miR-4653-3p target positions on the 3′-UTR.

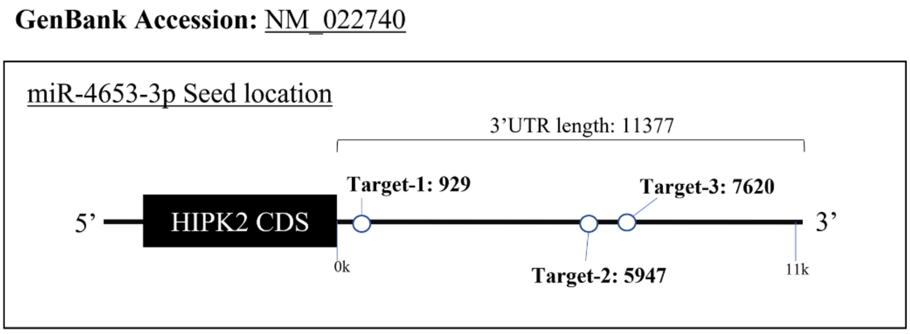

Supplementary Figure 2. Original chemiluminescent image of the capillary and charge data in the western blot analysis.

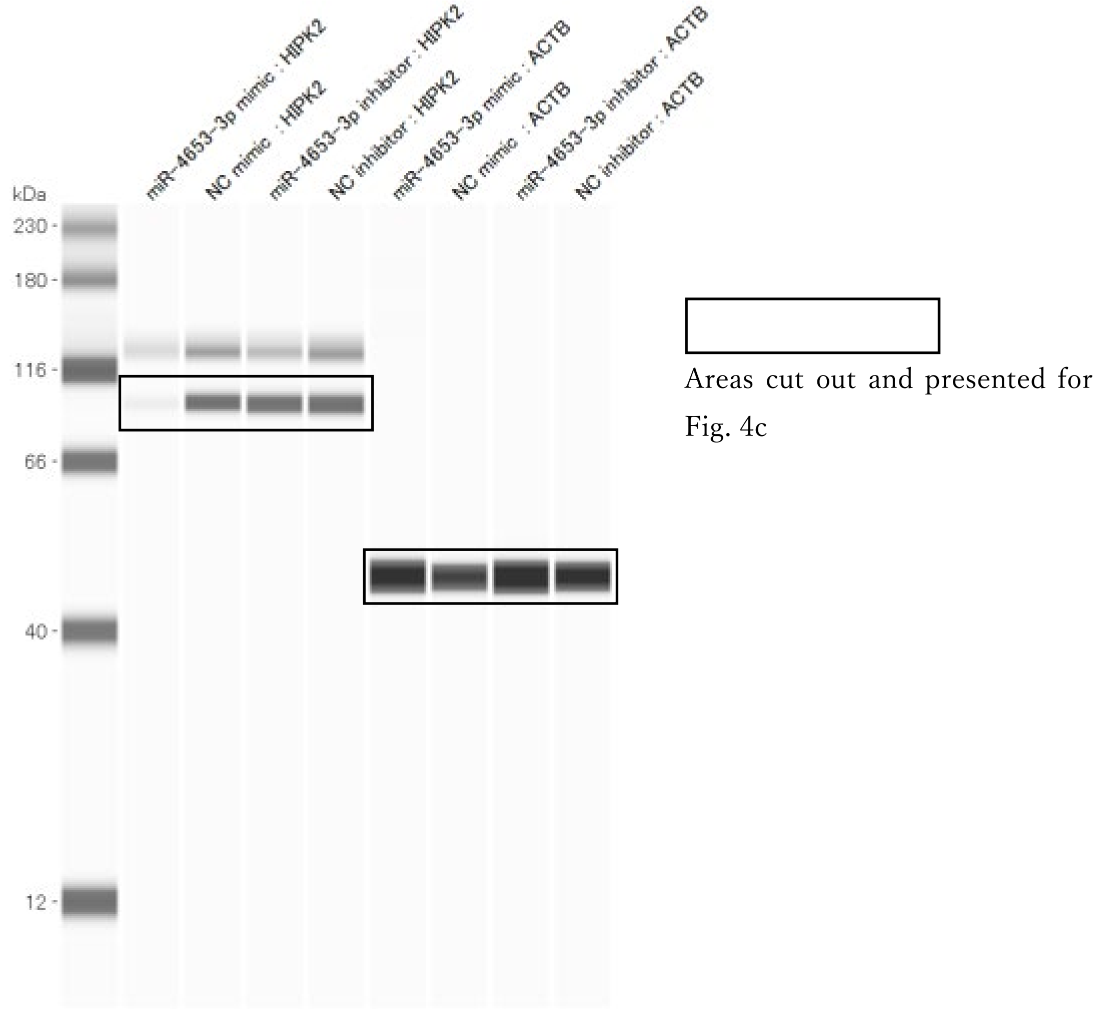

Supplementary Table 1a. Univariate and multivariate analyses of prognostic factors predicting the overall survival in patients with pancreatic ductal adenocarcinoma

	Univariate					Multivariate				
	HR	95% CI			*p*- value	HR	95% CI			*p* -value
Age > 65 years (vs. ≤ 65 years)	1.133	0.671	to	1.914	0.639	-				
Sex: Female (vs. male)	0.963	0.582	to	1.593	0.884	-				
Perineural invasion (vs. no)	5.077	1.235	to	20.863	**0.024**	n.e.				
Lymphatic involvement (vs. no)	3.714	1.161	to	11.874	**0.027**	n.e.				
Venous involvement (vs. no)	6.538	0.903	to	47.314	0.063	-				
pT: Primary tumor: pT3 (vs. pT1-2)	2.425	1.424	to	4.131	**0.001**	n.e.				
pN: Regional LN metastasis: pN1-2 (vs. pN0)	2.152	1.257	to	3.684	**0.005**	n.e.				
pM: Distant metastasis: pM1 (vs. pM0)	6.829	2.365	to	19.721	**<0.001**	5.269	1.813	to	15.313	**0.002**
Stage: IIA–IV (vs. IA–IB)	2.646	1.459	to	4.8	**0.001**	2.501	1.37	to	4.568	**0.003**
Histopathological grade: G2–3 (vs. G1)	1.087	0.656	to	1.802	0.746	-				
miR-4653-3p positive (vs. negative)	1.738	0.993	to	3.041	0.053	-				
HIPK2 negative (vs. positive)	0.557	0.321	to	0.966	**0.037**	n.e.				

HR, hazard ratio; CI, confidence interval; n.e., Variables not in the equation.

Supplementary Table 1b. Univariate and multivariate analyses of prognostic factors predicting the disease-specific survival in patients with pancreatic ductal adenocarcinoma

	Univariate					Multivariate				
	HR	95% CI			*p*- value	HR	95% CI			*p* -value
Age > 65 years (vs. ≤ 65 years)	1.154	0.676	to	1.969	0.6	-				
Sex: Female (vs. male)	0.964	0.578	to	1.608	0.888	-				
Perineural invasion (vs. no)	10.07	1.391	to	72.926	**0.022**	6.964	0.94	to	51.571	0.057
Lymphatic involvement (vs. no)	5.487	1.337	to	22.511	**0.018**	n.e.				
Venous involvement (vs. no)	24.087	0.539	to	1076.316	0.101	-				
pT: Primary tumor: pT3 (vs. pT1-2)	2.595	1.512	to	4.453	**<0.001**	n.e.				
pN: Regional LN metastasis: pN1-2 (vs. pN0)	2.223	1.284	to	3.848	**0.004**	n.e.				
pM: Distant metastasis: pM1 (vs. pM0)	7.012	2.421	to	20.306	**<0.001**	5.222	1.793	to	15.211	**0.002**
Stage: IIA–IV (vs. IA–IB)	2.806	1.52	to	5.18	**<0.001**	2.138	1.14	to	4.008	**0.018**
Histopathological grade: G2–3 (vs. G1)	1.095	0.655	to	1.831	0.73	-				
miR-4653-3p positive (vs. negative)	1.969	1.099	to	3.53	**0.023**	n.e.				
HIPK2 negative (vs. positive)	0.532	0.305	to	0.927	**0.026**	n.e.				

HR, hazard ratio; CI, confidence interval; n.e., Variables not in the equation.

Supplementary Table 2. Results of mRNA microarray.
MIA PaCa-2 cells transfected with miR-4653-3p mimic compared with negative control cells (|fold change| >2.0, p <0.05).

Gene_Symbol	Gene_ID	RefSeqAccession	Fold change (mimic/negative control)	Adjusted p-value
PLP2	5355	NM_002668	-9.232265	1.27127E-56
LRRN3	54674	NM_018334	-7.651053	1.32923E-16
APOO	79135	NM_024122	-7.529449	9.87191E-51
SLC44A1	23446	NM_080546	-7.261849	5.72167E-36
MOB3A	126308	NM_130807	-7.207636	4.94483E-36
ZP3	7784	NM_007155	-7.075344	3.58798E-22
RPL27A	6157	NM_000990	-5.467876	5.61909E-18
MXD4	10608	NM_006454	-5.312919	2.29769E-20
SSR1	6745	NM_003144	-5.280014	2.53353E-22
MXD4	10608	NM_006454	-5.186299	8.45435E-23
MYB	4602	NM_005375	-4.931653	1.94672E-18
KIAA1549	57670	NM_001164665	-4.813582	1.49436E-14
PSG3	5671	NM_021016	-4.801781	5.58378E-12
UACA	55075	NM_001008224	-4.650271	1.91252E-09
PLAGL2	5326	NM_002657	-4.634388	4.64988E-18
DLG5	9231	NM_004747	-4.472001	1.10153E-19
EOMES	8320	NM_005442	-4.461026	4.40507E-11
TCF7L1	83439	NM_031283	-4.404391	8.85298E-25
MLLT1	4298	NM_005934	-4.396842	2.83452E-17
SNAI2	6591	NM_003068	-4.329194	4.98012E-10
MLLT1	4298	NM_005934	-4.222486	2.13345E-28
SEPT8	23176	NM_015146	-4.208911	1.69413E-11
FAM171A1	221061	NM_001010924	-4.159357	1.76674E-21
BCL9	607	NM_004326	-4.137097	1.05305E-16
LOC646762	646762	NR_024278	-4.134648	8.52342E-18
TPRN	286262	NM_001128228	-4.056848	6.71742E-35
AP1S3	130340	NM_001039569	-4.007144	4.00108E-09
NCR3LG1	374383	NM_001202439	-4.004971	2.60775E-10
CADM2	253559	NM_001167674	-3.910583	6.68561E-06
PSG5	5673	NM_001130014	-3.879588	2.78689E-11
OXTR	5021	NM_000916	-3.852115	3.19071E-09
CXXC5	51523	NM_016463	-3.818359	1.64031E-20
GIT1	28964	NM_014030	-3.816602	3.60654E-08
NRM	11270	NM_007243	-3.749778	2.55821E-13

DGCR14	8220	NM_022719	-3.737215	1.4445E-10
TM4SF1-AS1	100874091	NR_109809	-3.73027	8.39091E-11
SCARNA11	677780	NR_003012	-3.72709	1.39389E-15
TM4SF1-AS1	100874091	NR_109809	-3.709618	6.74749E-11
GPR56	9289	NM_201525	-3.68578	1.39554E-17
PMP22	5376	NM_000304	-3.679847	6.38932E-15
SH3PXD2B	285590	NM_001017995	-3.668023	2.1562E-07
CTSC	1075	NM_148170	-3.656605	1.21552E-11
lnc-KCNQ2-1		XM_006723787	-3.640305	3.94118E-07
AP1S3	130340	NM_001039569	-3.640016	3.24611E-12
SORD	6652		-3.600355	9.5803E-07
VEPH1	79674	NM_024621	-3.586174	9.1898E-17
lnc-C12orf75-1			-3.52323	1.10391E-08
HSPA2	3306	NM_021979	-3.522658	2.1306E-06
LBHD1	79081	NM_024099	-3.496154	1.35804E-18
RNA5-8S5	100008587	NR_003285	-3.465714	5.73062E-32
lnc-GNA12-1			-3.447197	6.83197E-15
LINC00518	221718	NR_027793	-3.43036	1.33033E-09
KIRREL	55243	NM_018240	-3.417101	5.43552E-15
AHCYL2	23382	NM_015328	-3.404104	2.34443E-11
EIF4EBP2	1979	NM_004096	-3.391693	1.66979E-15
LOC100130930	100130930		-3.380228	1.29851E-06
ANXA11	311		-3.345899	3.68323E-05
OLIG1	116448	NM_138983	-3.317845	6.10473E-06
lnc-FAM3A-1			-3.308278	8.10408E-06
LOC255187	255187		-3.30406	2.14701E-08
SCARNA8	677776	NR_003009	-3.293717	1.41075E-17
LOC101927372	101927372	XR_244439	-3.29138	2.78718E-05
ABLIM2	84448	NM_032432	-3.284359	5.64428E-05
PDCD4-AS1	282997	NR_026932	-3.281201	6.86E-12
HIVEP3	59269	NM_024503	-3.280692	1.64101E-15
FAM155B	27112	NM_015686	-3.263846	4.42381E-13
LINC01468	101928687	NR_120641	-3.261667	1.12126E-10
PCDH9	5101		-3.253854	4.48559E-05
SCARNA1	677774	NR_002997	-3.250308	5.85111E-08
PML	5371	NM_033238	-3.250129	2.06658E-06
PITX1	5307	NM_002653	-3.248213	3.32667E-19
MRPL19	9801	NM_014763	-3.247045	3.12492E-10
LINC00163	727699	NR_033840	-3.21754	7.10639E-09

PIGS	94005	NM_033198	-3.214425	2.51559E-21
PDLIM5	10611	NM_006457	-3.19247	8.3469E-15
WIPF2	147179	NM_133264	-3.148734	6.66432E-14
SIPA1L3	23094	NM_015073	-3.145356	2.67335E-06
TRAK1	22906	NM_001042646	-3.136741	1.92478E-10
TGFBRAP1	9392	NM_004257	-3.127344	7.45921E-15
SNAP25	6616	NM_003081	-3.118851	7.2567E-12
GNPTG	84572	NM_032520	-3.112418	1.36134E-14
FRS2	10818	NM_001278351	-3.1107	1.66405E-08
PIGS	94005	NM_033198	-3.110416	5.02247E-06
LIMK1	3984	NM_002314	-3.102334	7.37839E-15
lnc-NPDC1-1			-3.08636	5.96959E-06
DLX4	1748	NM_138281	-3.070003	1.75759E-11
EEF2K	29904	NM_013302	-3.066993	2.81037E-09
PITX1	5307	NM_002653	-3.054044	2.2705E-21
PACSIN1	29993	NM_020804	-3.047111	1.97204E-13
GPR19	2842	NM_006143	-3.046958	1.66587E-05
SMG7	9887	NM_173156	-3.045185	1.14596E-08
ROR1	4919	NM_005012	-3.024793	2.60775E-10
MKI67	4288	NM_002417	-3.014588	1.34309E-12
SSR1	6745	NM_003144	-3.013707	9.77495E-20
KIRREL	55243	NM_018240	-3.002093	2.2305E-11
FAM19A5	25817	NM_015381	-2.996468	2.07245E-08
PLCD3	113026	NM_133373	-2.992781	3.0022E-05
CHST12	55501	NM_018641	-2.970434	1.18984E-12
MKI67	4288	NM_002417	-2.967682	2.54985E-10
SNORA1	677792	NR_003026	-2.939518	1.71456E-13
lnc-RP1-239B22.1.1-1			-2.927854	5.77773E-10
FAM117B	150864	NM_173511	-2.919792	8.78812E-10
BCR	613	NM_004327	-2.9115	1.72276E-09
EML4	27436	XM_005264268	-2.90973	4.11074E-19
PCNP	57092	NM_020357	-2.909404	7.73253E-13
CNP	1267	NM_033133	-2.880937	3.56371E-08
PRDX3	10935	NM_006793	-2.87208	1.88715E-07
TPCN1	53373	NM_001143819	-2.870455	6.14827E-13
BCR	613	NM_004327	-2.856213	1.03859E-11
CCDC73	493860	NM_001008391	-2.852399	0.00010393
PIK3R3	8503	NM_001303429	-2.850995	3.21561E-07
HIRA	7290	NM_003325	-2.837315	5.26674E-16

UBAP2	55833	NM_018449	-2.833809	1.07007E-13
RBBP4	5928	NM_005610	-2.833494	8.46856E-09
ZKSCAN1	7586	NM_001287054	-2.819722	2.56779E-12
WIPI2	26100	NM_015610	-2.818861	2.90936E-10
SNORA63	6043	NR_002586	-2.81279	2.6446E-16
lnc-NXPH2-2			-2.80908	0.000890634
GATAD2B	57459	NM_020699	-2.807643	5.77654E-07
FSTL1	11167	NM_007085	-2.801695	2.73356E-09
LRP3	4037	NM_002333	-2.800383	2.31558E-06
GATA4	2626	NM_002052	-2.798929	1.25202E-05
PDAP1	11333	NM_014891	-2.784459	5.69825E-06
ARHGAP26	23092	NM_015071	-2.779763	3.15183E-05
AIM2	9447	NM_004833	-2.769737	5.72379E-05
lnc-TARDBP-2			-2.766633	3.66989E-08
SBSN	374897	NM_001166034	-2.764238	0.000126257
BGLAP	632	NM_199173	-2.763259	3.05031E-07
CIZ1	25792	NM_001131015	-2.761686	4.33853E-07
NEK6	10783	NM_014397	-2.756932	2.35712E-09
PRR11	55771	NM_018304	-2.748813	2.97795E-12
TMEM115	11070	NM_007024	-2.7479	1.93021E-07
GINS1	9837	NM_021067	-2.745547	1.56392E-13
TXNDC17	84817	NM_032731	-2.742196	5.68453E-08
RPL28	6158	NM_001136134	-2.738994	1.2443E-15
SDPR	8436	NM_004657	-2.734752	1.69793E-11
ROR1	4919	NM_005012	-2.72804	1.16539E-06
RFT1	91869	NM_052859	-2.718815	1.47332E-06
KIAA1462	57608	NM_020848	-2.718286	4.2069E-08
NKAIN1	79570	NM_024522	-2.715696	5.19736E-08
BAMBI	25805	NM_012342	-2.712021	7.38315E-10
MFSD10	10227	NM_001120	-2.710844	5.3688E-10
RFX7	64864	NM_022841	-2.701647	7.66127E-09
SNORA2B	677794	NR_002951	-2.700572	1.62162E-12
DRGX	644168		-2.699626	0.000101803
MIB1	57534	NM_020774	-2.698863	7.31336E-07
ACTR1A	10121	NM_005736	-2.68881	3.03433E-12
PPAPDC3	84814	NM_032728	-2.687364	1.62607E-06
LINC00518	221718	NR_027793	-2.686932	0.000105325
ASF1B	55723	NM_018154	-2.686863	1.22595E-12
LOC340335	340335		-2.68228	5.93518E-06

lnc-FZD4-1			-2.676521	2.36105E-05
lnc-NKD2-3			-2.670633	6.83584E-07
ARHGAP5-AS1	84837	NR_027263	-2.670014	2.59425E-07
SNORA75	654321	NR_002921	-2.667452	5.31156E-08
NEURL1B	54492	NM_001142651	-2.663194	9.92777E-10
lnc-NKD2-3			-2.656366	2.83516E-09
PLAGL2	5326	NM_002657	-2.65266	2.60775E-10
lnc-ZNF484-1			-2.652281	4.6469E-05
LOC101928569	101928569	NR_104669	-2.644953	2.79268E-06
FSD1	79187	NM_024333	-2.644444	2.62045E-10
LOC100233156	100233156	NR_037871	-2.643014	0.023029234
NEXN	91624	NM_144573	-2.633723	6.98327E-05
SKIDA1	387640	NM_207371	-2.623442	0.000238142
POMGNT1	55624	NM_001243766	-2.620644	1.69413E-11
ZIC2	7546	NM_007129	-2.615689	0.000932122
RAB8B	51762	NM_016530	-2.614756	0.024143047
TK2	7084	NM_004614	-2.604471	4.72144E-07
SPSB1	80176	NM_025106	-2.59905	4.84458E-10
DST	667	XM_005249317	-2.59859	0.001769996
PXK	54899	NM_017771	-2.591674	3.94118E-07
SMAP1	60682	NM_001044305	-2.591659	6.90054E-09
GATA4	2626	NM_002052	-2.590059	6.13398E-07
ADCK3	56997	NM_020247	-2.587655	2.27631E-08
FLJ26086	440129		-2.58477	0.000442835
LOC100132077	100132077	NR_033937	-2.582485	1.24086E-05
ACOX1	51	NM_001185039	-2.57996	8.66294E-11
NQO2	4835	XR_249926	-2.579119	0.00165068
NF2	4771	NM_181832	-2.578979	1.9413E-22
TSHZ1	10194	NM_005786	-2.567271	5.93199E-07
POMGNT1	55624	NM_001243766	-2.565873	1.2264E-11
SMC1A	8243	NM_006306	-2.560368	3.14966E-07
TBX2	6909	NM_005994	-2.558247	3.59851E-12
PNRC1	10957	NM_006813	-2.557247	1.4026E-11
ITGB1BP1	9270	NM_004763	-2.55555	1.38142E-06
OBFC1	79991	NM_024928	-2.55376	0.000804112
AAK1	22848	NM_014911	-2.548603	7.00166E-11
TANC2	26115	NM_025185	-2.546469	1.07257E-08
FBXO33	254170	NM_203301	-2.540513	1.0726E-09
ALDH7A1	501	NM_001182	-2.538406	0.000133775

SNORA2A	677793		-2.533454	1.13391E-05
PCYOX1	51449	NM_016297	-2.532011	1.02654E-09
SNORA2A	677793	NR_002950	-2.525715	3.4594E-08
MARCH3	115123	NM_178450	-2.524832	0.001263092
GLRX5	51218	NM_016417	-2.523337	4.4568E-15
ILF3	3609	NM_004516	-2.522828	7.55047E-07
RNA18S5	100008588	NR_003286	-2.519474	8.34726E-42
PMF1-BGLAP	100527963	NM_001199662	-2.51398	0.000271433
NANOS1	340719	NM_199461	-2.513894	1.93591E-07
ZNF117	51351	NM_015852	-2.507884	5.28931E-05
ARL3	403	NM_004311	-2.505417	1.61463E-05
lnc-NUFIP2-1			-2.505337	0.005305417
TYSND1	219743	NM_173555	-2.501318	8.38153E-06
AMH	268	NM_000479	-2.500849	4.39505E-06
C17orf53	78995	NM_024032	-2.499996	0.000154878
SON	6651	NM_001291411	-2.498051	3.92783E-08
EPS8	2059	NM_004447	-2.494246	5.84598E-07
PDE3B	5140	NM_000922	-2.49347	0.000334946
CAV1	857	NM_001753	-2.491127	5.67932E-09
LSM3	27258		-2.485155	0.003586875
lnc-CEMP1-1			-2.484935	1.62083E-05
FLJ42627	645644	NR_024492	-2.481919	0.000151098
TAB3	257397	NM_152787	-2.481406	2.45974E-06
JAGN1	84522	NM_032492	-2.480555	8.82025E-10
EXTL3	2137	NM_001440	-2.477057	6.1824E-12
lnc-NUMB-2			-2.47545	9.22297E-05
BTC	685	NM_001729	-2.474784	0.000102242
BTG2	7832	NM_006763	-2.463152	3.64129E-10
DHRS2	10202	NM_182908	-2.462772	2.45462E-06
ALPK3	57538	NM_020778	-2.461795	0.000616743
ADCK1	57143	NM_020421	-2.45228	0.000207708
TBC1D14	57533	NM_020773	-2.448173	1.00255E-07
RAVER1	125950	NM_133452	-2.442247	7.23391E-07
EMB	133418	NM_198449	-2.441276	0.001304437
TGFBRAP1	9392	NM_004257	-2.440907	0.000353016
ADRB2	154	NM_000024	-2.440885	0.000154878
ST3GAL4-AS1	399972	NR_033839	-2.43947	0.000262303
COL13A1	1305	NM_080801	-2.43936	0.00639932
lnc-ATXN3L-2			-2.437551	2.56708E-05

CPLX1	10815	NM_006651	-2.432882	6.27561E-05
LINC01468	101928687	NR_120641	-2.426041	0.002222902
LPCAT1	79888	NM_024830	-2.421339	2.71551E-05
ZNRF3	84133	NM_001206998	-2.417917	3.57971E-11
MKI67	4288	NM_002417	-2.417644	1.00695E-09
BZW1	9689	NM_014670	-2.41644	3.97611E-08
MARK2	2011	NM_001039469	-2.411217	1.13257E-06
NFE2	4778	NM_006163	-2.407053	0.000251206
GSK3A	2931	NM_019884	-2.404059	2.09805E-14
LINC01420	550643	NR_015367	-2.401149	4.49612E-07
SRCAP	10847	NM_006662	-2.397685	0.001311451
TATDN2	9797	NM_014760	-2.395568	3.59133E-07
ADAL	161823	NM_001012969	-2.395179	0.000132691
HEY1	23462	NM_001040708	-2.394279	0.000143638
SLC16A9	220963	NM_194298	-2.387987	0.000249224
UBL7	84993	NM_032907	-2.387731	1.4759E-08
RUNX1	861	NM_001001890	-2.387611	9.56376E-10
PIK3R2	5296	NM_005027	-2.386579	2.55964E-15
RGS5	8490	NM_003617	-2.381378	4.19753E-10
XPR1	9213	NM_004736	-2.38112	2.51791E-05
LFNG	3955	NM_001040167	-2.379829	1.25515E-05
LINC01468	101928687	NR_120641	-2.379512	0.006204235
MICB	4277	NM_005931	-2.374947	6.5907E-07
CAV1	857	NM_001753	-2.370935	6.65147E-07
PEG10	23089	NM_001040152	-2.369445	0.000172487
MRPL17	63875	NM_022061	-2.368418	2.35778E-08
WIPI2	26100	NM_001033518	-2.364728	2.34455E-12
TTL	150465	NM_153712	-2.36466	1.28809E-16
PDE4A	5141	NM_006202	-2.362718	8.87052E-08
PRKAR2B	5577	NM_002736	-2.359142	3.11905E-10
LOC101928249	101928249	XR_428024	-2.35857	6.43054E-06
RHOQ	23433	NM_012249	-2.357284	5.25599E-10
EDN1	1906	NM_001955	-2.34476	0.003156875
ABHD14A	25864	NM_015407	-2.341947	1.22392E-09
PPARA	5465	NM_005036	-2.340741	0.00337155
IFITM10	402778	NM_001170820	-2.339755	3.91448E-05
ARHGEF17	9828	NM_014786	-2.338856	6.89536E-08
ABHD14A	25864	NM_015407	-2.337923	3.98185E-10
EML4	27436	XM_005264267	-2.33309	3.36662E-09

CARD9	64170	NM_052813	-2.333048	3.73587E-06
LINC01420	550643	NR_015367	-2.332148	9.73661E-09
EDC3	80153	NM_025083	-2.331503	4.66821E-07
FNTB	2342	NM_002028	-2.329717	2.79268E-06
NRP2	8828	NM_201264	-2.32904	0.001134485
LMBR1	64327	NM_022458	-2.326064	0.000107003
SNORA12	677800		-2.32247	0.000155476
KCNMB4	27345	NM_014505	-2.320224	1.13419E-05
ELL2	22936	NM_012081	-2.31926	0.000395285
ISCA2	122961	NM_194279	-2.318497	1.18267E-09
FBXO25	26260		-2.316376	0.005959394
SSH2	85464	NM_001282130	-2.315356	9.42383E-07
DIEXF	27042	NM_014388	-2.314084	0.003420339
MSL1	339287	NM_001012241	-2.312795	5.9854E-08
HOXB5	3215	NM_002147	-2.308788	2.0903E-06
SNORA80E	677823	NR_002974	-2.308475	3.26472E-10
PKP2	5318	NM_004572	-2.307949	5.74866E-05
DENND5B	160518	NM_144973	-2.304303	2.39245E-06
lnc-CHID1-1			-2.301909	0.004646595
UBOX5	22888	NM_014948	-2.301519	5.87178E-05
XLOC_l2_008203			-2.296375	0.001190947
PGS1	9489	NM_024419	-2.296326	0.000157812
BAD	572	NM_004322	-2.295915	5.55759E-09
BCORL1	63035	NM_021946	-2.295369	1.45813E-07
ZHX3	23051	NM_015035	-2.294874	8.26934E-09
HIPK2	28996	NM_022740	-2.291512	0.002701668
LOC254896	254896	NR_046173	-2.290645	2.27912E-06
AKAP5	9495	NM_004857	-2.289229	0.003390289
GATA4	2626	NM_002052	-2.280937	0.003494008
CEP68	23177		-2.278017	0.001266661
NRP1	8829	NM_001024629	-2.277098	0.002329279
CCDC28A	25901	NM_015439	-2.273699	1.25999E-07
PTCD3	55037		-2.273057	2.81326E-06
SLC26A10	65012	NM_133489	-2.271366	0.003371916
PRKAA1	5562	NM_206907	-2.270601	7.34236E-05
LRIG2	9860	XR_426743	-2.26773	2.52083E-05
SNAP25-AS1	100131208	NR_040710	-2.266171	0.004010076
PCYOX1	51449	NM_016297	-2.265413	5.14773E-08
PAICS	10606	NM_001079525	-2.265192	1.09579E-10

CYP4Z1	199974	NM_178134	-2.264423	0.008854759
PNRC1	10957	NM_006813	-2.262886	1.40608E-05
TOB1	10140	NM_005749	-2.257066	6.98461E-08
XLOC_l2_011798			-2.256856	0.001852066
LOC100506476	100506476	NR_109995	-2.2561	0.004553991
EFNA5	1946	NM_001962	-2.255612	0.000181285
ICAM3	3385	NM_002162	-2.253075	1.35617E-07
TNFAIP1	7126	NM_021137	-2.25282	3.21561E-07
VPS26A	9559	NM_004896	-2.248613	9.18571E-06
lnc-APLN-1			-2.246424	1.44895E-05
lnc-ZFYVE26-1			-2.246032	0.047515723
UBQLN4	56893		-2.245462	4.04109E-07
VMA21	203547	NM_001017980	-2.245277	1.13573E-11
NR2F1	7025	NM_005654	-2.24456	1.14883E-16
FLJ42627	645644	NR_024492	-2.244043	0.000372138
VANGL1	81839	NM_138959	-2.242586	2.73451E-09
SOWAHD	347454	NM_001105576	-2.242301	0.000394815
LOC100505478	100505478	NM_001199233	-2.241926	2.2722E-05
RAB6B	51560	NM_016577	-2.241691	0.001041665
GYS1	2997	NM_002103	-2.241067	1.43607E-08
lnc-COPS7B-1			-2.240328	0.001063487
TSPAN14	81619	NM_030927	-2.238697	2.2967E-05
GPM6B	2824	NM_001001995	-2.236744	0.015229266
SNX24	28966	NM_014035	-2.236344	1.50295E-07
MECP2	4204	NM_004992	-2.23567	1.37817E-05
SNORA38B	100124536	NR_003706	-2.235141	6.49806E-08
SCARNA14	692149	NR_004388	-2.231959	1.45233E-06
WWC2	80014	NM_024949	-2.230324	0.000864774
CAV3	859	NM_001234	-2.229993	0.001060566
ZHX3	23051	NM_015035	-2.229337	2.77657E-06
lnc-IFIT2-1			-2.228119	3.70558E-05
lnc-ALDH1A3-1			-2.228103	0.003246849
ISOC2	79763	NM_024710	-2.226204	4.11313E-09
MRE11A	4361	NM_005590	-2.224033	5.04644E-06
KCNJ14	3770	NM_013348	-2.222769	2.84107E-07
UBE2Q2	92912	NM_173469	-2.222491	2.40892E-07
lnc-RHBDD1-1			-2.22088	0.010527526
TUBD1	51174	NM_016261	-2.22015	7.14207E-07
SNORA51	677831	NR_002981	-2.219458	2.87826E-08

TIMP2	7077	NM_003255	-2.214481	1.36134E-14
lnc-UCK1-1			-2.214287	0.003154204
DOCK5	80005	NM_024940	-2.213812	0.01803782
TM9SF4	9777	NM_014742	-2.213685	3.35681E-07
lnc-GOLPH3L-1			-2.212327	0.001552005
MVB12B	89853	NM_033446	-2.211979	0.001852458
MBP	4155	NM_001025101	-2.211439	0.001576236
GATS	352954	NR_028038	-2.210521	4.82846E-05
RNF169	254225	NM_001098638	-2.207376	3.16425E-05
IPO5	3843	NM_002271	-2.205551	1.71502E-06
SNORA79	677845	NR_003021	-2.201422	8.89784E-07
MIR600HG	81571	NR_026677	-2.199684	0.007652484
SCARNA18	677765	NR_003139	-2.199646	7.77379E-09
MXI1	4601	NM_130439	-2.197298	9.51271E-08
TMEM37	140738	NM_183240	-2.196828	3.04989E-05
CYP4Z1	199974	NM_178134	-2.19674	0.008477787
lnc-LAMA5-1			-2.19418	5.5525E-05
CRTAP	10491	NM_006371	-2.187535	0.012105866
SHC3	53358	NM_016848	-2.18724	5.31544E-07
EXTL1	2134	NM_004455	-2.185109	0.00757421
ULK2	9706	NM_014683	-2.184869	8.94739E-05
OSR2	116039	NM_053001	-2.182702	0.003351456
SYCE2	256126	NM_001105578	-2.182666	0.000887574
C14orf1	11161	NM_007176	-2.18244	1.91339E-05
OGFRL1	79627	NM_024576	-2.182158	1.45149E-05
lnc-RP4-604K5.1.1-2			-2.177431	0.014788665
MSRB2	22921	NM_012228	-2.176808	0.000693652
CALB2	794	NM_001740	-2.176522	8.21355E-07
SIX4	51804	NM_017420	-2.173873	3.99226E-05
CCSER2	54462	NM_001284241	-2.171323	2.86869E-06
APOE	348	NM_001302688	-2.169429	4.5877E-05
SUB1	10923	NM_006713	-2.169029	2.5197E-05
CLDN5	7122	NM_001130861	-2.167582	0.00108801
MVB12B	89853	NM_033446	-2.161473	4.65022E-07
FOXRED2	80020	NM_024955	-2.161442	8.39475E-07
CENPN	55839	NM_001100624	-2.161076	1.32935E-06
SDHC	6391		-2.159779	0.014488868
DIO2	1734	NM_013989	-2.157442	8.09361E-06
BRICD5	283870	NM_182563	-2.156739	0.000255809

TGFBI	7045	NM_000358	-2.154858	6.95336E-07
FAT3	120114	NM_001008781	-2.154001	2.2484E-07
NAV1	89796	NM_020443	-2.152407	0.001072494
HS1BP3	64342	NM_022460	-2.151611	2.67222E-07
lnc-SUSD1-1			-2.151386	0.003769187
ZNF480	147657	NM_144684	-2.151275	6.73863E-05
lnc-CDH2-3			-2.147239	0.000311777
CASC10	399726	NM_001010911	-2.146483	1.01867E-06
LINC00565	100861555	NR_047495	-2.145765	0.01059138
IL6ST	3572	NM_001190981	-2.145658	4.48989E-05
RIMBP3	85376	NM_015672	-2.14486	3.36157E-05
KIF23	9493	NM_138555	-2.14281	2.35778E-08
UBAP2	55833	NM_018449	-2.141254	4.48341E-05
TCF3	6929	NM_003200	-2.136158	3.40648E-09
FAM78A	286336	NM_033387	-2.135923	3.20377E-05
JADE2	23338	NM_015288	-2.135417	7.25337E-06
DDTL	100037417	NM_001084393	-2.130221	0.000510736
BAZ2A	11176	NM_013449	-2.129355	0.000446605
GM2A	2760	NM_000405	-2.128739	0.001650988
COL10A1	1300	NM_000493	-2.123592	0.003528685
DHRS4-AS1	55449	NR_023921	-2.123348	5.63198E-06
TFAP2A	7020		-2.121558	0.001895223
AHR	196	NM_001621	-2.115541	0.003106093
CDC25A	993	NM_001789	-2.115286	1.20981E-05
PDCD4	27250	NM_145341	-2.114779	1.90122E-05
GTPBP1	9567	NM_004286	-2.112089	1.11582E-05
SNORA6	574040	NR_002325	-2.111226	1.92265E-08
BZW1	9689	NM_014670	-2.111076	0.001156987
LUZP6	767558	NM_001128619	-2.108967	1.78496E-05
COA1	55744	NM_018224	-2.108686	1.98836E-06
lnc-GINS2-1			-2.108613	0.003145983
ST5	6764	NM_005418	-2.108111	3.83712E-05
PI4KB	5298	NM_001198773	-2.106173	5.91604E-06
CCSER2	54462	NM_018999	-2.104952	8.0966E-05
LOC100129534	100129534	NR_024489	-2.104871	8.0788E-05
FOXP4	116113	NM_001012426	-2.103841	0.000165061
PTP4A2	8073	NM_080391	-2.103587	7.52162E-08
PIANP	196500	NM_153685	-2.102514	0.000349144
SHOX2	6474	NM_003030	-2.099663	4.52415E-10

TMEM132B	114795	NM_052907	-2.099313	0.023932139
POM121	9883	NM_172020	-2.098842	2.49106E-06
lnc-MAP3K9-3			-2.094611	0.000493305
IGF2BP2	10644	NM_006548	-2.094096	1.98183E-06
C7orf26	79034	NM_024067	-2.092087	1.10906E-06
LINC00476	100128782	NR_023390	-2.089855	0.02328286
PPAP2A	8611	NM_176895	-2.089653	7.50292E-07
lnc-CDH16-2			-2.089375	0.000782299
LINC00908	284276		-2.087569	0.000769106
UBN2	254048	NM_173569	-2.087531	0.000631037
CDKL1	8814	NM_004196	-2.085827	0.001367285
SIMC1	375484	XM_005265909	-2.08578	0.002002523
F8A1	8263	NM_012151	-2.082093	2.30334E-08
NOP14-AS1	317648	NR_015453	-2.082	0.004021688
GNL1	2794	NM_005275	-2.080524	0.000255201
LY6G5B	58496	NM_021221	-2.077583	0.004175374
HPS1	3257	NM_000195	-2.074214	0.000910876
MED18	54797	NM_017638	-2.073617	0.002523479
MEF2B	100271849	NM_001145785	-2.070772	4.99338E-06
lnc-TAF15-1			-2.066264	0.008473137
MYO10	4651	NM_012334	-2.065874	2.89332E-05
TRAPPC1	58485	NM_021210	-2.065814	5.61731E-10
PI4KB	5298	NM_002651	-2.065508	7.20956E-06
C19orf70	125988	NM_205767	-2.064662	6.01381E-08
SAP25	100316904	NM_001168682	-2.064355	5.35965E-06
EPHA8	2046	NM_001006943	-2.063963	0.012595682
SAP30L	79685	NM_024632	-2.063562	0.002365988
CD44	960	NM_000610	-2.063319	2.40713E-14
SNORA62	6044	NR_002324	-2.059827	7.88528E-07
XLOC_l2_011649			-2.059792	0.000103094
LINC01204	101927528	NR_104645	-2.059758	0.010029056
LOC283911	283911		-2.058064	1.37744E-05
SNORA4	619568	NR_002588	-2.058055	8.18783E-06
C7orf61	402573	NM_001004323	-2.056312	0.001020523
CYP2U1	113612	NM_183075	-2.055837	0.002014566
PSMG4	389362	NM_001135750	-2.05507	0.002724983
LOC100131048	100131048		-2.053286	0.009109157
LOC285178	285178		-2.052868	1.86682E-06
CCDC80	151887	NM_199511	-2.05117	0.011551795

SPG7	6687		-2.049142	0.000437859
FZD1	8321	NM_003505	-2.048146	0.000974399
CYBRD1	79901	NM_024843	-2.04705	0.008580178
SLC6A6	6533	NM_003043	-2.046639	0.009889143
LRIG2	9860	XR_426743	-2.045482	0.021835021
ZNF362	149076	NM_152493	-2.042355	1.53364E-05
ALDH7A1	501	NM_001182	-2.042078	7.46302E-05
LRFN1	57622	NM_020862	-2.041393	0.000355611
LOC100129550	100129550	NR_024618	-2.041254	0.001037106
SCARNA4	677771	NR_003005	-2.040994	0.006382936
CRYBG3	131544	NM_153605	-2.040741	0.003834188
TMEM50B	757	NM_006134	-2.040329	5.52702E-05
BRINP3	339479	NM_199051	-2.040232	6.80279E-05
QDPR	5860	NM_000320	-2.039922	2.52621E-05
DSEL	92126	NM_032160	-2.039869	0.014372698
LOC729680	729680		-2.039772	0.004593296
DYNC1LI2	1783	NM_006141	-2.039302	8.53398E-08
SNORA27	619499	NR_002575	-2.038689	5.15013E-07
SOBP	55084	NM_018013	-2.038168	0.003425082
LOC91450	91450	NR_026998	-2.037799	0.023428462
FAM124A	220108	NM_145019	-2.03714	0.017903148
LOC100506674	100506674	NR_109862	-2.037136	0.000256203
NCOA1	8648	NM_147233	-2.036722	0.000199269
CERK	64781	NM_022766	-2.035978	8.14421E-06
MEF2B	100271849	NM_001145785	-2.034796	0.000346652
FAM192A	80011	NM_024946	-2.033841	4.19658E-05
lnc-FBXW5-1			-2.03189	0.002172782
GPR125	166647	NM_145290	-2.030952	0.000179998
C2CD2	25966		-2.030358	0.026904476
VSTM2L	128434	NM_080607	-2.030155	2.36381E-05
NEUROD2	4761	NM_006160	-2.028829	0.008523256
TTC39A	22996	NM_001080494	-2.028623	0.001044244
MYPN	84665	NM_032578	-2.028393	0.000793233
SNORD32B	692092	NR_003049	-2.026654	7.88495E-05
VAMP4	8674	NM_003762	-2.025754	0.004527916
AHNAK	79026	NM_001620	-2.024021	4.27088E-05
SMAD6	4091	NM_005585	-2.022484	0.000504481
DIO2	1734	NM_013989	-2.018545	0.018141732
LOC143286	143286		-2.018387	0.004076185

RASA2	5922	XM_006713719	-2.017425	0.018217667
SHC3	53358	NM_016848	-2.016096	6.11834E-05
APOBR	55911	NM_018690	-2.015286	0.004589226
C2orf68	388969	NM_001013649	-2.014957	0.00754903
SEMA3C	10512	NM_006379	-2.014067	0.008627125
DKFZP564C152	26120		-2.014059	5.52313E-05
ABR	29	NM_021962	-2.013497	8.79783E-06
DKK1	22943	NM_012242	-2.010832	0.000311777
UTP15	84135	NM_032175	-2.009439	0.000391874
PDIA2	64714	NM_006849	-2.007928	2.83277E-05
IL6ST	3572	NM_001190981	-2.007903	0.00463626
LOC100128361	100128361	NR_036505	-2.004552	0.018871532
SNORD32A	26819	NR_000021	-2.004512	1.19993E-05
UTRN	7402	NM_007124	-2.002733	8.27873E-05
ATP2A1	487	NM_173201	-2.000501	0.024282538
MLEC	9761		2.001113	0.000205101
ZNF25	219749	NM_145011	2.001302	0.004580244
CREB3L3	84699	NM_032607	2.001556	0.014440331
OASL	8638	NM_003733	2.002477	0.000689213
LSM14A	26065	NM_015578	2.003327	3.4382E-08
DCK	1633	NM_000788	2.004832	3.56241E-06
TWF2	11344	NM_007284	2.006211	0.002998355
lnc-CCDC71L-1			2.006317	0.008282413
DHDH	27294	NM_014475	2.007448	0.001314531
OSTM1	28962	NM_014028	2.007677	0.000227764
ZDHHC21	340481	NM_178566	2.008573	0.014020808
CXADR	1525	NM_001338	2.009033	0.022194268
lnc-RP11-293M10.1.1-1			2.012402	0.006921964
SERPINB8	5271	NM_001031848	2.012544	0.000109537
GBA	2629	NM_001005741	2.013018	0.000552999
AKR1E2	83592	NM_001271025	2.013302	0.00926237
NECAP1	25977	NM_015509	2.013513	7.53974E-07
KCNG3	170850	NM_133329	2.015181	0.003433746
PTK2B	2185	NM_173174	2.016349	0.006325012
SLC25A20	788	NM_000387	2.016669	0.000256568
LOC101927787	101927787	NR_125944	2.01798	0.002453043
ADAT2	134637	NM_001286259	2.018204	0
HPSE	10855	NM_006665	2.018401	0.002279941
CA2	760	NM_000067	2.019619	0.002180268

WDR26	80232	NM_025160	2.020085	1.45865E-08
LOC101927851	101927851		2.020212	4.36371E-09
GPX8	493869	NM_001008397	2.020744	2.05385E-07
SCYL3	57147	NM_020423	2.021286	0.000132492
ARFRP1	10139	NM_003224	2.021887	0.001653943
CASP1	834	NM_033292	2.023207	8.14103E-06
RACGAP1	29127	NM_013277	2.023476	1.27043E-07
LINC01063	101929769	XR_246052	2.024317	0.003600734
B4GALT1	2683	NM_001497	2.024606	0.019396869
ACADSB	36	NM_001609	2.02509	0.000339983
PAXIP1-AS1	202781	NR_028090	2.025619	0.000654655
ZNF669	79862	NM_024804	2.025835	0.008083226
MIAT	440823	NR_003491	2.026191	0.005168329
NAA35	60560	NM_024635	2.02657	8.89784E-07
C8orf31	286122	NM_173687	2.027016	0.000271234
KLK6	5653	NM_001012964	2.027688	1.77588E-12
CSRNP1	64651	NM_033027	2.03046	0.006412895
SPATA2L	124044	NM_152339	2.030854	0.009119551
CANX	821	NM_001746	2.031496	0.001480386
JUNB	3726	NM_002229	2.03177	0.000939239
HS1BP3	64342		2.032667	0.001162107
CFAP45	25790	NM_012337	2.03314	0.040638425
PLCXD1	55344	NM_018390	2.033362	0.000781933
CCDC125	202243	NM_176816	2.033722	1.06674E-05
TVP23B	51030	NM_016078	2.037123	2.94227E-09
WBP4	11193	NM_007187	2.038255	1.18478E-07
FAM188B	84182	NM_032222	2.038881	0.007103425
SNORD116-19	727708	NR_001290	2.038924	0.00232877
SUPT3H	8464	NM_181356	2.039923	0.011239383
GTPBP2	54676	NM_019096	2.040435	0.012411779
RABEPK	10244		2.040806	7.4651E-09
CDC23	8697		2.041455	4.76386E-05
N6AMT1	29104		2.0419	0.002434771
DZIP3	9666	NM_014648	2.042116	1.4967E-05
ORAI3	93129	NM_152288	2.043668	0.001206596
ZNF684	127396	NM_152373	2.043958	0.000151098
LINC01003	100128822	NR_027387	2.044237	4.98904E-06
SERPINB1	1992	NM_030666	2.045448	1.43949E-07
FGFBP3	143282	NM_152429	2.045549	0.002722629

PAQR3	152559	NM_001040202	2.04585	1.204E-06
FOS	2353	NM_005252	2.046875	3.70558E-05
MSH5	4439	NM_002441	2.047684	0.000901468
CRB3	92359	NM_139161	2.049522	0.02935839
ATP9A	10079	NM_006045	2.050132	0.003725488
SLC30A7	148867	NM_133496	2.051293	0.000458445
lnc-UQCRFS1-9			2.053158	0.000244486
LINC00482	284185	NR_038080	2.054045	0.019009891
SNX8	29886		2.05478	0.000420982
LOC399815	399815	NR_027282	2.05488	0.001725546
FAM216A	29902	NM_013300	2.055416	1.79213E-09
ANP32E	81611	NM_030920	2.058112	1.82647E-06
lnc-UQCRFS1-9			2.058179	7.07536E-05
HMGN1	3150	NM_004965	2.058324	4.20086E-14
P2RY2	5029	NM_176072	2.059484	0.001948584
PGGT1B	5229	NM_005023	2.060092	7.40702E-06
CCDC125	202243	NM_176816	2.060833	1.43747E-05
OPLAH	26873	NM_017570	2.063633	4.48341E-05
IL10RA	3587	NM_001558	2.064132	0.017990655
lnc-FSCN2-1			2.064792	0.001715887
lnc-PMM2-6			2.06529	1.70155E-07
ANK2	287	NM_001148	2.065964	0.013764461
LOC101928880	101928880	NR_110944	2.066731	0.002217981
RDM1	201299	NM_145654	2.069122	0.002940975
CCNT2	905	NM_058241	2.071205	5.09325E-06
PGPEP1	54858	NM_017712	2.074532	2.98268E-05
UBXN8	7993	NM_005671	2.074774	0.000172982
GCH1	2643	NM_001024071	2.076638	0.000826168
SMAD2	4087	NM_001003652	2.077816	0.000271281
PHLDA2	7262	NM_003311	2.077876	5.62516E-10
RASSF5	83593	NM_182663	2.078208	0.002837342
MAT2B	27430	NM_182796	2.07918	2.6877E-08
FLCN	201163	NM_144606	2.079355	5.71808E-05
FKBP7	51661	NM_181342	2.080319	0.00135078
NR6A1	2649	NM_033334	2.080616	0.018650494
AGTPBP1	23287	NM_015239	2.080986	2.9514E-06
lnc-SNURF-1			2.081751	0.002701668
ANP32E	81611	NM_030920	2.081995	5.04552E-07
B3GNT5	84002	NM_032047	2.082221	0.000574131

FUOM	282969	NM_198472	2.082503	0.000578004
CXCL8	3576	NM_000584	2.083381	2.39245E-06
RAB3D	9545	NM_004283	2.084288	0.007888829
UBXN8	7993	NM_005671	2.084802	1.0409E-05
MPRIP	23164		2.085417	2.74715E-05
PARP11	57097	NM_020367	2.085713	0.003805202
MNT	4335	NM_020310	2.087831	0.003515931
IVNS1ABP	10625	NM_006469	2.088168	1.38142E-06
ACAD11	84129	NM_032169	2.091856	0.00092679
WDR12	55759	NM_018256	2.091934	1.6211E-07
ARFRP1	10139	NM_001267549	2.091953	1.61884E-06
MSMO1	6307	NM_006745	2.093253	2.80824E-06
APOBEC3G	60489	NM_021822	2.09455	0.002626586
ELOVL6	79071	NM_024090	2.094645	0.000524481
PNMA5	114824	NM_052926	2.094836	4.21795E-05
SELT	51714	NM_016275	2.094897	6.56202E-08
WNT6	7475	NM_006522	2.09544	0.005754301
ZRANB1	54764	NM_017580	2.097387	0.000973921
KIRREL2	84063	NM_199180	2.097873	6.18667E-06
SLC33A1	9197	NM_001190992	2.0983	5.96146E-06
ARHGAP19	84986	NM_001204300	2.100104	0.001361436
ING3	54556	NM_198267	2.100537	0.00012241
LINC00673	100499467	NR_036488	2.100788	5.07698E-05
DPYSL5	56896	NM_020134	2.101163	2.52356E-05
XPOT	11260	NM_007235	2.101285	0
LINC01184	644873	NR_015360	2.102156	0.000784744
AGPAT4	56895	NM_020133	2.106381	0.008362303
LINC00540	100506622	NR_103810	2.107566	0.00018448
TMEM144	55314	NM_018342	2.108408	0.000578004
RARRES3	5920	NM_004585	2.109107	1.07E-05
OSBPL11	114885	NM_022776	2.109687	8.88898E-08
ACBD3	64746	NM_022735	2.11079	1.00255E-07
MEIG1	644890	NM_001080836	2.113667	0.001407011
FCRLB	127943	NM_001002901	2.114899	0.000119907
CCDC71L	168455	NM_175884	2.115568	1.3721E-09
ENTPD8	377841	NM_001033113	2.115702	0.038692041
LOC101929448	101929448	NR_125925	2.115813	0.022634124
PHTF1	10745	NM_006608	2.117803	1.13136E-05
MAPK11	5600	NM_002751	2.120907	0.000166843

TXNRD1	7296	NM_001261445	2.121507	4.85558E-06
LINC01547	84536	NR_027128	2.126446	0.002562094
SLC35G2	80723	NM_025246	2.128273	2.71953E-06
MESDC1	59274	NM_022566	2.129049	4.26339E-07
C10orf54	64115	NM_022153	2.129081	0.000373214
SLC16A5	9121	NM_004695	2.130053	4.1701E-10
NCKAP1	10787	NM_205842	2.132054	8.12075E-09
GPCPD1	56261	NM_019593	2.132449	1.84572E-05
DPY19L1	23333	NM_015283	2.134565	5.09311E-06
RNF170	81790	NM_030954	2.135642	1.29502E-07
C4orf29	80167	NM_001039717	2.135671	5.64428E-05
TDG	6996	NM_003211	2.136257	1.82909E-09
FAM27C	100132948	NR_027421	2.138155	2.96996E-05
TMEM55A	55529	NM_018710	2.139023	7.5742E-07
LOC100128317	100128317	NR_126025	2.139397	0.000788293
PNMA6A	84968	NM_032882	2.139966	0.002202331
FGF21	26291	NM_019113	2.14146	0.010477918
IMPA1	3612	NM_005536	2.142045	3.96086E-08
LINC00662	148189		2.144167	3.82955E-06
SLC19A1	6573	NM_194255	2.144748	3.02016E-05
F2R	2149		2.146348	4.56913E-06
FGD5-AS1	100505641	NR_046251	2.146717	0.000498625
TMPO	7112	NM_003276	2.147414	2.12634E-10
APOL6	80830	NM_030641	2.150258	0.000461856
FAM184A	79632	NM_024581	2.150533	0.002178302
TTN-AS1	100506866	NR_038271	2.152047	0.006600707
RRAS2	22800	NM_012250	2.152592	2.59496E-07
CCDC125	202243	NM_176816	2.152835	6.73863E-05
BCL10	8915	NM_003921	2.156663	0.000404874
RAD51AP1	10635	NM_006479	2.156719	4.33185E-12
lnc-SEZ6L2-1			2.15878	2.60775E-10
ERCC1	2067	NM_202001	2.158842	1.18482E-06
PIGN	23556	NM_176787	2.159023	2.06315E-05
FAM135A	57579	NM_020819	2.159479	4.01203E-06
SECISBP2	79048	NM_001282690	2.159659	0.000682562
PIK3C2B	5287	NM_002646	2.160069	6.3807E-06
MRAP2	112609	NM_138409	2.160653	0.019126004
LINC00963	100506190	NR_038955	2.160777	0.000291926
ATXN1L	342371	NM_001137675	2.161846	1.0895E-06

LGALS8	3964	NM_006499	2.164936	5.44535E-07
NAT6	24142	NM_012191	2.165808	0.002972009
RWDD4	201965	NM_152682	2.168327	5.28931E-05
NUDT18	79873	NM_024815	2.168927	0.000215064
ABCA5	23461	NM_018672	2.169261	0.001311986
EDEM1	9695	NM_014674	2.169379	3.45665E-07
FAM129A	116496	NM_052966	2.170389	7.65237E-07
SLC35A5	55032	NM_017945	2.170403	1.24939E-06
VEGFA	7422	NM_001025366	2.174858	7.71017E-07
PPP1R15A	23645	NM_014330	2.175043	1.70782E-05
CCNB3	85417	NM_033031	2.176829	0.010401
TTN-AS1	100506866	NR_038272	2.178059	8.18856E-05
NSF	4905	NM_006178	2.178708	5.22223E-07
HRASLS5	117245	NM_054108	2.180067	3.71302E-13
ARHGEF2	9181	NM_004723	2.180486	2.51796E-05
UBXN8	7993	NM_005671	2.181439	3.53005E-05
FAM131A	131408	NM_144635	2.18326	0.000527622
LMBRD2	92255	NM_001007527	2.1845	0.000979942
LMCD1	29995	NM_014583	2.186519	0.003770245
PNMA6A	84968	NM_032882	2.186786	6.25171E-05
PAIP1	10605	NM_006451	2.187793	8.11629E-09
RRAS2	22800	NM_012250	2.189338	3.26398E-08
IL18R1	8809	NM_003855	2.189741	1.14874E-07
YTHDF2	51441	NM_001173128	2.189795	4.36876E-06
PFKFB2	5208	NM_001018053	2.192077	0.000172982
APOL4	80832	NM_030643	2.192145	0.001597309
CD9	928	NM_001769	2.19349	1.69413E-11
CLHC1	130162	NM_152385	2.194264	0.001335588
NHP2L1	4809	NM_005008	2.194358	1.2438E-05
F11R	50848	NM_016946	2.194887	1.87278E-07
LINC01122	400955	NR_033873	2.194912	1.1758E-10
DCTD	1635	NM_001012732	2.196865	3.21561E-07
ZNF684	127396	NM_152373	2.197266	0.001525471
AREG	374	NM_001657	2.19885	3.87849E-12
ZNF727	442319	XR_242241	2.199591	0.000136511
ZBTB20-AS1	100131117	NR_038993	2.199596	3.91974E-12
ATP6V0A1	535	NM_005177	2.200367	3.09901E-08
CLCN4	1183	NM_001830	2.200538	0.000426139
ODF2L	57489	NM_001184765	2.20178	5.24273E-05

DDX58	23586	NM_014314	2.204668	0.000155972
MRPS35	60488	NM_021821	2.205037	2.26501E-10
MAMSTR	284358	NM_182574	2.205669	0.000540578
ZBTB43	23099	NM_014007	2.210354	9.29502E-07
FGD5-AS1	100505641	NR_046251	2.211442	0.000234697
POLDIP3	84271	NM_032311	2.211755	5.44535E-07
CRYGS	1427	NM_017541	2.212507	0.002464066
NSUN3	63899	NM_022072	2.21251	5.26713E-05
CHST14	113189	NM_130468	2.213192	0.00810912
TXNRD3	114112	NM_052883	2.213564	2.48114E-06
HRH1	3269	NM_001098213	2.21514	0.000840806
AGPAT4	56895	NM_020133	2.216099	0.001283665
EME2	197342		2.217265	0.005337651
BCAT1	586	NM_005504	2.217379	1.47423E-05
lnc-GPR183-2			2.217614	0.000419301
CPEB4	80315	NM_030627	2.217655	1.35731E-06
LCA5	167691	NM_181714	2.218716	0.000151098
DUSP2	1844	NM_004418	2.218752	0.000373251
IFT88	8100	NM_175605	2.219232	8.65167E-06
AP4B1	10717	NM_006594	2.220653	0.001512365
CTF1	1489	NM_001330	2.221049	3.50121E-06
SETD7	80854	NM_030648	2.221774	5.84025E-06
ZNF571	51276	NM_016536	2.223652	0.000345987
LOC101930611	101930611	XR_250546	2.225246	0.006090432
PLEKHG2	64857	NM_022835	2.22571	2.52848E-06
SLC35A3	23443	NM_012243	2.230274	2.45966E-07
FAM27C	100132948	NR_027421	2.231276	0.000601561
CARS	833	NM_001014437	2.231645	1.94889E-05
SLC35B3	51000	NM_015948	2.232353	9.46478E-09
GRAMD1B	57476	NM_001286563	2.232973	3.56012E-06
SESN2	83667	NM_031459	2.239295	2.44437E-06
ARAF	369	NM_001256197	2.240025	2.62615E-05
RNF41	10193	NM_194358	2.246363	9.24146E-05
SLC16A3	9123	NM_001042422	2.24758	0
RBM34	23029	NM_001161533	2.247715	0.001071566
CEBPB	1051	NM_005194	2.248175	5.79137E-13
LINC00963	100506190		2.249293	0.000462042
SLC38A6	145389	NM_153811	2.249538	3.31441E-09
ICA1	3382	NM_004968	2.250338	0.000202899

SLC10A7	84068	NM_001300842	2.251825	0.00108902
NUPR1	26471	NM_001042483	2.252139	1.69653E-08
BCL2L2	599	NM_004050	2.253424	4.86083E-07
KPNA5	3841	XM_006715474	2.253619	0.000224314
LOC143666	143666	NR_026967	2.254915	0.000864774
RBM18	92400	NM_033117	2.257148	2.54154E-08
UBR3	130507	NM_172070	2.257569	2.03617E-06
lnc-C14orf166B-3			2.257883	0.001718361
PFKFB4	5210	NM_004567	2.25822	0.000231144
TMEM110	375346	NM_198563	2.2595	8.7755E-07
ERCC1	2067	NM_202001	2.259921	2.18073E-09
HMGN1	3150	NM_004965	2.260795	7.73253E-13
AP4B1	10717	NM_006594	2.261417	6.76911E-06
ZNF697	90874	NM_001080470	2.261697	0.001435037
EOGT	285203	NM_173654	2.264352	1.6934E-05
F3	2152	NM_001993	2.267272	0.000205101
LOC101929709	101929709		2.267817	0.001161466
LOC101927200	101927200	XR_244180	2.269323	4.78679E-05
CHD2	1106	NM_001042572	2.270116	0.000909915
MAFG	4097	NM_032711	2.271553	1.62083E-05
ZEB1-AS1	220930	NR_024284	2.272057	6.2036E-07
C17orf51	339263	NM_001113434	2.272418	6.67637E-06
ZRSR2	8233		2.273	0.003161494
lnc-UQCRFS1-9			2.280944	0.00185421
RPGR	6103	NM_000328	2.282272	1.37028E-06
CEACAM1	634	NM_001712	2.285777	2.86869E-06
AREG	374	NM_001657	2.291276	0
PLK3	1263	NM_004073	2.29189	7.79345E-07
BTBD9	114781	NM_052893	2.292347	0.003952979
lnc-TMEM88B-1			2.29733	0.01322936
ZBTB20	26137	NM_015642	2.29945	0.00506622
CREB5	9586	NM_182898	2.299844	2.50532E-05
LCOR	84458	NM_032440	2.301281	5.34746E-06
UBA6-AS1	550112	NR_015439	2.305091	4.54195E-06
THOC7-AS1	100874039	NR_104326	2.305755	2.71568E-07
VAMP2	6844	NM_014232	2.306351	0.000161464
KDM4C	23081	NM_001146695	2.307005	0.00113095
HUS1	3364	NM_004507	2.307886	0.000108627
FAM46C	54855	NM_017709	2.311073	0.000267073

C11orf65	160140	NM_152587	2.312422	5.67735E-05
CYB561D1	284613	NM_001134404	2.313623	4.04167E-07
ENO1-AS1	100505975	NR_038351	2.313782	0.000746195
HSD17B4	3295	NM_000414	2.317142	6.15878E-11
LIPG	9388	NM_006033	2.319506	2.60059E-06
HKDC1	80201	NM_025130	2.320684	3.04112E-09
THAP9-AS1	100499177	NR_034075	2.322378	0.001731204
SQRDL	58472	NM_021199	2.325615	5.34804E-11
EPAS1	2034	NM_001430	2.328053	2.74839E-05
SNORD116-11	100033423	NR_003326	2.328237	0.002152774
HMGB3	3149	NM_001301228	2.328417	2.2309E-09
MAPK9	5601	NM_001135044	2.332786	4.21008E-05
TFPI2	7980	NM_006528	2.332875	0.009182077
RPH3AL	9501	NM_006987	2.334618	0.002018221
CXCL2	2920	NM_002089	2.338493	5.1243E-09
IL18RAP	8807	NM_003853	2.34053	6.08929E-07
MOSPD2	158747	NM_152581	2.340804	6.51994E-09
TMEM263	90488	NM_152261	2.340997	2.82147E-08
NHP2L1	4809	NM_005008	2.343195	5.132E-13
COMMD10	51397	NM_016144	2.345029	6.97906E-11
RNFT1	51136	NM_016125	2.345265	5.37762E-08
TNS4	84951	NM_032865	2.345617	0.002791578
IFRD1	3475	NM_001007245	2.351207	6.64059E-11
EAF1	85403	NM_033083	2.351949	8.33665E-10
DLG1	1739	NM_004087	2.352104	1.55708E-10
AATK	9625	NM_001080395	2.354465	1.04932E-07
SFMBT1	51460	NM_016329	2.354982	6.16165E-07
LOC102723385	102723385	NR_119379	2.355301	0.004070762
LOC101928880	101928880	NR_110944	2.359147	6.08768E-05
PPP1R15B	84919	NM_032833	2.36051	1.11576E-10
CNOT6L	246175	NM_001286790	2.36302	4.30923E-06
SSH1	54434	NM_001161331	2.366092	6.93454E-06
VGF	7425	NM_003378	2.36753	1.52553E-11
ZNF25	219749	NM_145011	2.369563	7.13506E-06
C5orf34	375444	NM_198566	2.370024	3.65038E-09
CCDC15	80071	NM_025004	2.37288	1.18913E-07
ZBTB20	26137	NM_001164343	2.373286	0.002864489
GABARAPL1	23710	NM_031412	2.376941	7.50188E-05
ANKRD37	353322	NM_181726	2.379124	6.98327E-05

LOC102724002	102724002	XR_424156	2.381429	2.71868E-07
VLDLR	7436	NM_003383	2.381674	4.36876E-06
PTPRH	5794	NM_002842	2.386275	5.22223E-07
TFEB	7942	NM_007162	2.38738	5.58442E-06
CD274	29126	NM_014143	2.38793	0.001707121
KIAA0232	9778	NM_014743	2.392146	3.4331E-10
ULBP1	80329	NM_025218	2.393915	0.001948584
PYROXD1	79912	NM_024854	2.396736	4.68772E-06
NCOA7	135112	NM_181782	2.402129	6.15075E-10
ATHL1	80162	NM_025092	2.406891	3.02222E-05
ELOVL7	79993	NM_024930	2.406977	3.18518E-05
XBP1	7494	NM_005080	2.406988	0
LOC101927285	101927285	NR_110219	2.408287	3.75707E-09
TP53INP2	58476	NM_021202	2.412338	5.20778E-06
HES4	57801	NM_021170	2.416939	2.30207E-13
RSG1	79363	NM_030907	2.417795	3.02381E-05
NMD3	51068	NM_015938	2.420448	4.20086E-14
lnc-BBC3-1			2.420474	5.06637E-05
lnc-CCDC33-1		XM_006720697	2.420592	0.001910568
SH3BP5	9467	NM_004844	2.422163	8.43016E-05
CASP9	842	NM_001229	2.422493	1.58548E-08
MB	4151	NM_203377	2.422527	0.002459641
lnc-OR4M2-7			2.422845	1.18813E-07
KCNK5	8645	NM_003740	2.423096	1.03829E-10
EGR4	1961	NM_001965	2.427016	7.08935E-06
KLF2	10365	NM_016270	2.43212	0
lnc-WNT1-2			2.433137	6.81073E-06
HIF1A	3091	NM_181054	2.43381	0
ATP9A	10079	NM_006045	2.433957	9.02233E-08
CXADR	1525	NM_001338	2.439811	2.37555E-06
COMMD8	54951	NM_017845	2.440971	8.89174E-12
APOL6	80830	NM_030641	2.446702	0.005551354
LINC00662	148189	NR_027301	2.449617	8.7755E-07
AMMECR1	9949	NM_001171689	2.449861	3.75707E-09
ABHD3	171586	NM_138340	2.452365	1.17754E-07
THAP9-AS1	100499177	NR_034075	2.453805	6.91482E-05
SNRPD1	6632		2.455135	5.45181E-06
ZBTB43	23099	NM_014007	2.45678	7.90831E-06
lnc-DNTTIP2-1			2.459229	3.97258E-09

FRRS1	391059	NM_001013660	2.460517	0.000562567
ZBTB46	140685	NM_025224	2.462998	3.09008E-05
TUBE1	51175	NM_016262	2.464998	3.4384E-09
C22orf39	128977	NM_173793	2.465404	5.21412E-07
MCTP1	79772	NM_024717	2.465779	0.002808591
CBR4	84869	NM_032783	2.469435	3.59805E-07
RIMS3	9783	NM_014747	2.469941	0.003176638
TLR1	7096	NM_003263	2.47411	0.000522078
FBXO4	26272	NM_012176	2.475207	5.24715E-10
FLJ46906	441172	NR_033896	2.476833	0.000442835
lnc-ZNF843-2			2.477559	0.000391966
PRIM2	5558	NM_000947	2.478725	0.000153526
KIF4A	24137	NM_012310	2.480394	3.5378E-09
EDEM3	80267	NM_025191	2.483501	1.26858E-09
UGCG	7357	NM_003358	2.491544	7.96791E-07
WNT11	7481	NM_004626	2.492519	0.003817464
TMEM194B	100131211	NM_001142645	2.496264	0.000279491
NSD1	64324	NM_022455	2.496588	0.000484474
ABCG1	9619	NM_207627	2.499764	3.8092E-06
SLC22A4	6583	NM_003059	2.504417	0.00028479
ERICH2	285141	NM_001290030	2.505334	0.004678758
C9orf91	203197	NM_153045	2.505368	1.94651E-07
IL21R	50615	NM_181078	2.51177	0.00018162
KIF4A	24137	NM_012310	2.516051	1.27788E-06
ERICH2	285141	NM_001290031	2.51665	0.001372756
ZNF641	121274	NM_152320	2.521763	0.000379511
ATP6V1A	523	NM_001690	2.522627	1.94432E-13
ENC1	8507	NM_003633	2.529212	0
PTP4A3	11156	NM_032611	2.531142	5.81329E-05
FLJ43315	644316	NR_033856	2.534748	1.73842E-06
TMEM41B	440026	NM_001165030	2.535642	4.74142E-07
MAFG	4097	NM_002359	2.536774	7.4745E-11
LINC00659	100652730	NR_046224	2.541607	4.18447E-05
RHOB	388		2.542186	1.88803E-07
HGSNAT	138050	NM_152419	2.547039	7.42509E-10
ASNS	440	NM_001673	2.554108	0
PIM3	415116	NM_001001852	2.563537	0
BEX2	84707	NM_001168399	2.565286	6.11192E-10
JUND	3727	NM_001286968	2.567079	2.24077E-06

MOSPD1	56180	NM_019556	2.568688	4.36371E-09
SCOC	60592	NM_032547	2.569353	6.28916E-12
CEND1	51286	NM_016564	2.572707	2.50339E-07
KBTBD8	84541	NM_032505	2.583845	0.000924319
C4orf33	132321	NM_173487	2.58445	1.39323E-05
ULBP1	80329	NM_025218	2.58468	5.42022E-12
lnc-USP7-1			2.58668	0.000115411
MAFF	23764	NM_012323	2.586941	1.96494E-07
SOCS6	9306	NM_004232	2.588318	1.89699E-08
FGD5-AS1	100505641	NR_046254	2.588945	2.74704E-05
MAVS	57506	NM_020746	2.591725	2.03786E-10
KLHDC7B	113730	NM_138433	2.592721	2.81215E-06
ADD2	119	NM_017482	2.595377	1.25169E-06
EPT1	85465	NM_033505	2.596958	5.80015E-05
JDP2	122953	NM_130469	2.59773	5.24715E-10
LINC00162	378825	NR_024089	2.604547	1.63131E-08
GAD1	2571	NM_013445	2.613066	5.40646E-06
MCF2L-AS1	100289410	NR_034002	2.616548	2.85684E-05
CD97	976	NM_078481	2.618869	0
LOC100506797	100506797	XR_112377	2.619759	0.00042068
ZFP36	7538	NM_003407	2.620709	4.26199E-09
EHF	26298	NM_012153	2.624239	4.63096E-10
CREM	1390	NM_183013	2.624461	9.43437E-10
CCDC82	79780	NM_024725	2.625323	1.22392E-09
FAM151B	167555	NM_205548	2.632108	0.000463933
JKAMP	51528	NM_001284201	2.632847	5.11781E-06
YTHDF2	51441	NM_016258	2.633436	1.94432E-13
CSNK1A1	1452		2.636255	2.10595E-10
XLOC_l2_008130			2.643772	2.08931E-07
NXPE3	91775	NM_001134456	2.644653	0.000627336
LONRF1	91694	NM_152271	2.658537	3.56605E-08
PUS10	150962	NM_144709	2.660825	3.21754E-05
PER3	8863	NM_016831	2.662022	1.42649E-06
HS1BP3	64342		2.663245	1.00752E-07
C1D	10438	NM_006333	2.663282	1.61419E-11
MAGIX	79917	NM_024859	2.668211	3.00212E-05
PTK6	5753	NM_005975	2.6689	4.63462E-05
NES	10763	NM_006617	2.670118	3.54492E-06
UPP1	7378	NM_001287426	2.676373	4.00966E-09

ZC3H6	376940	NM_198581	2.681492	2.61536E-05
C18orf32	497661	NM_001035005	2.682321	9.3957E-09
NUPL1	9818		2.701078	1.07234E-10
C1D	10438	NM_006333	2.707509	1.59904E-07
ZBTB41	360023	NM_194314	2.722049	1.33033E-09
GOSR1	9527	NM_001007024	2.723299	2.48982E-09
CARF	79800	NM_024744	2.724278	9.84635E-05
LINC00963	100506190		2.732921	6.94251E-06
NPB	256933	NM_148896	2.733595	7.66494E-10
DNAJC5	80331	NM_025219	2.734429	4.93775E-09
GRPEL2	134266	NM_152407	2.736102	0
TTC39B	158219	NM_152574	2.739505	5.20271E-09
RARA-AS1	101929693	NR_110861	2.741314	2.49106E-06
RCAN1	1827	NM_004414	2.743078	0
LINC00162	378825	NR_024089	2.743994	8.9334E-12
IPPK	64768	NM_022755	2.744784	1.18741E-07
XLOC_l2_014504			2.751359	2.53481E-09
RNASE4	6038	NM_001282192	2.755915	1.08593E-08
KLHL28	54813	NM_017658	2.757435	4.02105E-08
SUPT16H	11198	NM_007192	2.757995	3.00924E-13
SLC7A6OS	84138	NM_032178	2.758484	2.60775E-10
TBC1D7	51256	NM_016495	2.765454	1.27893E-12
SLC35F5	80255		2.767603	4.85172E-06
NKX6-3	157848	NM_152568	2.782716	2.81215E-06
lnc-RP11-17M16.1.1-1		XM_006722594	2.783265	2.66192E-05
POMK	84197	NM_032237	2.783713	0.000100358
RHOB	388		2.78475	6.13278E-09
OSBPL6	114880	NM_032523	2.789249	2.12126E-10
ELF4	2000	NM_001421	2.792253	1.16514E-11
RPGR	6103	NM_000328	2.801013	0.000392575
SQSTM1	8878	NM_003900	2.80197	0
LOC102723673	102723673	XR_424639	2.803152	8.20444E-06
BIRC3	330	NM_001165	2.805287	6.45913E-11
LATS2	26524	NM_014572	2.823048	1.71317E-11
SMPDL3A	10924	NM_006714	2.837975	5.39622E-06
OTUD4	54726	NM_001102653	2.838054	1.81299E-10
RBM12	10137	NM_006047	2.838623	2.303E-07
FAR2	55711	NM_018099	2.84932	4.74804E-07
HBS1L	10767	NM_001145207	2.853836	7.35087E-08

GID8	54994	NM_017896	2.855238	4.20086E-14
PDIK1L	149420	NM_001243533	2.859527	6.32582E-05
SPRYD7	57213		2.868737	1.78575E-06
SLC1A4	6509	NM_003038	2.86933	3.44039E-05
EWSR1	2130	NM_013986	2.878004	3.71302E-13
CLGN	1047	NM_004362	2.885132	6.42289E-10
LINC00662	148189		2.888163	2.75334E-09
SPATA2L	124044	NM_152339	2.893509	8.16337E-14
TRUB1	142940	NM_139169	2.898211	8.16337E-14
PI4K2B	55300	NM_018323	2.90286	0
CCAT1	100507056	NR_108049	2.915407	1.75586E-11
TDG	6996	NM_003211	2.921276	1.27893E-12
RAB21	23011		2.927799	8.8448E-10
BCAP29	55973	NM_018844	2.928125	1.62162E-12
TUBA4A	7277	NM_006000	2.929285	0.000628289
TM7SF3	51768	NM_016551	2.93895	7.71433E-11
LOC344887	344887	NR_033752	2.944447	6.52148E-10
SMC2	10592	NM_001042550	2.944678	4.70258E-11
FGD5-AS1	100505641	NR_046251	2.951017	1.94432E-13
EPB41	2035	NM_203342	2.953996	0.000155568
DCP2	167227	NM_152624	2.959504	0
PHTF1	10745	NM_006608	2.967744	1.40856E-08
lnc-UQCRFS1-7			2.972214	8.0923E-12
FBXL17	64839		3.002201	4.1358E-06
DDIT4	54541	NM_019058	3.00746	0
CEBPG	1054	NM_001806	3.009123	0
FLJ43315	644316	NR_033856	3.012338	2.98543E-09
MPZL3	196264	NM_198275	3.015901	2.26585E-09
ARL5B	221079	NM_178815	3.019542	3.02932E-08
PRR3	80742		3.039242	2.07482E-06
LOC340340	340340	XR_252283	3.042571	2.73356E-09
SNCG	6623	NM_003087	3.069417	6.3034E-11
TMEM198	130612	NM_001005209	3.070957	2.42141E-06
LINC00525	84847	NR_038407	3.074091	8.27126E-12
ULBP2	80328	NM_025217	3.081336	3.57753E-10
LOC284561	284561	XR_110828	3.099111	6.01136E-08
CTH	1491	NM_001902	3.104324	0
DOK4	55715	NM_018110	3.107402	8.00116E-08
CANX	821	NM_001746	3.109352	1.10886E-10

UNC5B	219699	NM_170744	3.114913	2.19572E-10
lnc-ARRDC3-1			3.130854	7.24469E-09
TYW3	127253	NM_138467	3.132375	8.90837E-07
DNAJC6	9829	NM_001256864	3.136821	4.23745E-06
ARL5A	26225	NM_012097	3.139163	5.79137E-13
IER3	8870	NM_003897	3.151271	0
TRMT10A	93587	NM_152292	3.15353	3.7576E-09
PDIK1L	149420	NM_152835	3.16026	4.20086E-14
IER3	8870	NM_003897	3.169019	0
lnc-ACOT9-1			3.175386	9.34287E-06
MAP1LC3B	81631	NM_022818	3.198765	0
MAPK6	5597	NM_002748	3.201147	0
ATF3	467	NM_001040619	3.205132	0
TUBE1	51175		3.205619	1.43648E-05
LINC00662	148189		3.210204	9.33441E-06
YWHAB	7529	NM_003404	3.223323	0
BMF	90427	NM_001003940	3.22455	8.40033E-07
AP1AR	55435	NM_018569	3.226906	2.03101E-09
SREK1IP1	285672	NM_173829	3.234154	4.97319E-08
CHAC1	79094	NM_024111	3.235126	0
ZNF678	339500	NM_178549	3.249043	9.2592E-10
PTGES	9536	NM_004878	3.25802	2.80727E-09
TNFRSF6B	8771	NM_003823	3.273688	3.20388E-11
DDX60	55601	NM_017631	3.274194	1.50584E-07
lnc-ACER2-1			3.279053	2.63981E-09
TPK1	27010	NM_022445	3.281437	3.23194E-10
LOC102724910	102724910		3.289434	0
ARHGEF16	27237	NM_014448	3.296408	4.40278E-10
CHAC1	79094	NM_024111	3.301362	2.98604E-09
ZNF641	121274	NM_152320	3.305487	5.93375E-07
TLR6	10333	NM_006068	3.307301	2.24763E-05
GLI1	2735	NM_005269	3.315936	1.73945E-07
ARHGAP19	84986	NM_032900	3.32112	1.01572E-06
CD274	29126	NM_014143	3.351706	4.78327E-09
TMEM182	130827	NM_144632	3.357763	4.11467E-09
BHLHE40	8553	NM_003670	3.361743	2.09279E-12
SERP1	27230	NM_014445	3.393111	0
lnc-AC007405.7.1-1			3.394931	1.27389E-07
SMIM13	221710	NM_001135575	3.416317	0

MMD	23531	NM_012329	3.420398	9.73169E-11
TSC22D3	1831	NM_004089	3.435324	0
TM4SF19	116211	NM_138461	3.436356	5.87399E-09
BBC3	27113	NM_014417	3.448484	0
EGR1	1958	NM_001964	3.466137	0
IFIH1	64135	NM_022168	3.473129	3.72094E-10
ALDH1A3	220	NM_000693	3.482264	0
ETF1	2107	NM_004730	3.494507	0
CTGF	1490	NM_001901	3.499652	0
FAM3C	10447	NM_014888	3.503614	0
XLOC_l2_005692			3.508993	0
PMAIP1	5366	NM_021127	3.512355	0
CACUL1	143384	NM_153810	3.527815	1.59866E-13
CA13	377677	NM_198584	3.530849	1.2681E-09
TNFRSF6B	8771	NM_003823	3.537976	0
SMIM14	201895	NM_174921	3.59489	8.16337E-14
IGIP	492311	NM_001007189	3.603203	7.05488E-10
MAP1B	4131	NM_005909	3.607805	8.93969E-07
ZBTB7B	51043	NM_001252406	3.610773	5.01183E-08
UHMK1	127933	NM_175866	3.623351	1.66951E-09
MCOLN3	55283	NM_018298	3.62493	6.43094E-10
STRA6	64220	NM_001142620	3.63045	0
LIF	3976	NM_002309	3.658221	0
PGPEP1	54858	NM_017712	3.748605	8.70864E-07
RNASEL	6041	NM_021133	3.913896	1.93069E-09
DNMT3A	1788	NM_175630	3.927215	3.39157E-09
EPT1	85465	NM_033505	3.932637	0
ATAD1	84896	NM_032810	3.944592	0
IPPK	64768	NM_022755	3.956941	9.41756E-13
CSF3	1440	NM_000759	4.006292	3.32029E-12
lnc-DNASE1L3-1			4.009896	0
TRIB3	57761	NM_021158	4.030829	0
PSMG3-AS1	114796	NR_027329	4.068468	2.83516E-09
TRANK1	9881	NM_014831	4.069378	9.80605E-09
TM4SF19	116211	NM_138461	4.12296	1.22595E-12
ACVR2B	93	NM_001106	4.181808	1.10943E-12
RAB39B	116442	NM_171998	4.229137	5.99039E-11
SLC22A18AS	5003	NM_007105	4.269344	0
ZC3H6	376940	NM_198581	4.271913	2.60775E-10

TRIM23	373	NM_001656	4.287752	3.00924E-13
lnc-PMM2-5			4.325376	0
ATF3	467	NM_001040619	4.357662	3.12492E-10
TRMT10A	93587	NM_152292	4.359484	3.43928E-10
TLR3	7098	NM_003265	4.370306	2.64061E-11
DDIT3	1649	NM_004083	4.39714	0
NARS2	79731	NM_024678	4.540386	0
GBP3	2635	NM_018284	4.556653	5.20415E-12
RC3H2	54542		4.572215	0
TPMT	7172	NM_000367	4.575245	0
FUT1	2523	NM_000148	4.612099	0
SLC7A11	23657	NM_014331	4.65331	0
SMIM13	221710	NM_001135575	4.736027	0
UHMK1	127933	NM_175866	4.961436	0
RAB26	25837	NM_014353	5.090921	0
MNAT1	4331	NM_002431	5.188449	0
HIPK1	204851	NM_198268	5.304745	0
TRIML2	205860	NM_173553	5.645029	0
CXCL5	6374	NM_002994	6.049565	0
LINC00659	100652730	NR_046224	6.764034	0
PRSS30P	124221	NR_026864	7.258892	0
RRM2	6241	NM_001034	7.567009	0
C3orf52	79669	NM_024616	8.40204	0
AMMECR1	9949	NM_015365	8.532593	0
CACUL1	143384	NM_153810	9.622434	0
GDF15	9518	NM_004864	11.054186	0

Supplementary Table 3. HIPK2 expression and clinicopathological parameters

Total number of cases (n = 90)	HIPK2 expression Low (n = 18) n (%)	High (n = 72) n (%)	p- value
**Age**			
≤ 65 years (n = 32)	6 (18.8)	26 (81.2)	0.826
> 65 years (n = 58)	12 (20.7)	46 (79.3)	
**Gender**			
Male (n = 43)	7 (16.3)	36 (83.7)	0.399
Female (n = 47)	11 (23.4)	36 (76.6)	
**Primary tumor (pT)**			
pT1-pT2 (n = 61)	14 (23)	47 (77)	0.31
pT3 (n = 29)	4 (13.8)	25 (86.2)	
**Regional lymph node metastasis (pN)**			
pN0 (n = 41)	7 (17.1)	34 (82.9)	0.525
pN1 (n = 49)	11 (22.4)	38 (77.6)	
**Distant metastasis (pM)**			
pM0 (n = 86)	17 (19.8)	69 (80.2)	0.597
pM1 (n = 4)	1 (25)	3 (75)	
**Stage**			
Stage IA-IB (n = 32)	6 (18.8)	26 (81.3)	0.826
Stage IIA–IV (n = 58)	12 (20.7)	46 (79.3)	
**Perineural invasion**			
No (n = 9)	1 (11.1)	8 (88.9)	0.426
Yes (n = 81)	17 (21)	64 (79)	
**Lymphatic involvement**			
No (n = 11)	1 (9.1)	10 (90.9)	0.305
Yes (n = 79)	17 (21.5)	62 (78.5)	
**Venous involvement**			
No (n = 6)	0	6 (100)	0.251
Yes (n = 84)	18 (21.4)	66 (78.6)	
**Tumor grade**			
G1 (n = 44)	10 (22.7)	34 (77.3)	0.527
G2 or G3 (n = 46)	8 (17.4)	38 (82.6)	
